# Bromodomain-containing protein 4 (BRD4) as an epigenetic regulator of fatty acid metabolism genes and ferroptosis

**DOI:** 10.1038/s41419-022-05344-0

**Published:** 2022-10-29

**Authors:** Minghua Yang, Ke Liu, Pan Chen, Hongyi Zhu, Junjie Wang, Jun Huang

**Affiliations:** 1 Department of Pediatrics, The Third Xiangya Hospital, Central South University, Changsha, 410013 Hunan Province P. R. China; 2 Department of Ophthalmology, The Second Xiangya Hospital, Central South University, Changsha, 410011 Hunan Province P. R. China; 3 Hunan Cancer Hospital and The Affiliated Cancer Hospital of Xiangya School of Medicine, Central South University, Changsha, 410013 Hunan Province P. R. China; 4 Department of Gastroenterology, The Second Xiangya Hospital, Central South University, Changsha, 410011 Hunan Province P. R. China; 5 Department of Orthopedics, The Second Xiangya Hospital, Central South University, Changsha, 410011 Hunan Province P. R. China

**Keywords:** Single-molecule biophysics, Gene silencing

## Abstract

Reprogramming lipid metabolism is considered a fundamental step in tumourigenesis that influences ferroptosis. However, molecular mechanisms between lipid metabolism and ferroptosis remain largely unknown. Results from the drug screening of 464 inhibitors (for 164 targets) applied to ferroptosis cells indicated that 4 inhibitors targeted bromodomain-containing protein 4 (BRD4) significantly inhibiting erastin-induced ferroptosis. Functional studies proved that the loss of BRD4 weakened oxidative catabolism in mitochondria, protecting cells from the excessive accumulation of lipid peroxides. Mechanism research revealed that the transcriptional levels of fatty acid metabolism-related genes (HADH, ACSL1 and ACAA2) participating in the β-oxidation of fatty acids (FAO) and polyunsaturated fatty acids (PUFAs) synthesis depended on the activity of super-enhancers (SEs) formed by BRD4 and HMGB2 in their promoter regions. Conclusively, this study demonstrated that BRD4 was indispensable for fatty acid metabolism based on its epigenetic regulatory mechanisms and affecting erastin-induced ferroptosis, providing a new theoretical reference for understanding the relationship between lipid metabolism and ferroptosis deeply.

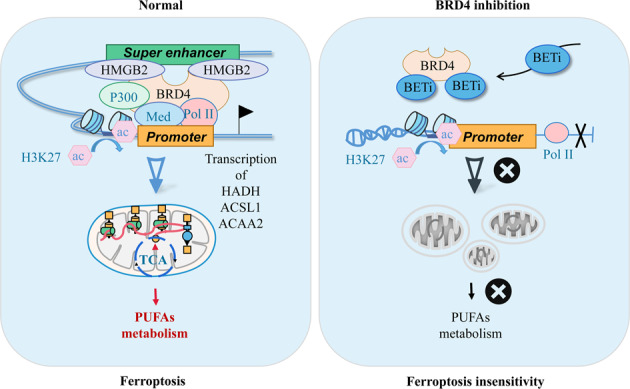

## Introduction

Ferroptosis is a programmed cell death process driven by iron-dependent lipid peroxidation [1]. Therefore, exploring the regulatory mechanisms of ferroptosis is of great significance in developing anti-tumour strategies, particularly for eradicating tumours resistant to conventional treatments [2]. Asides from cysteine aspartic acid protease (Caspase) dependent apoptosis and mixed-lineage kinase domain-like protein (MLKL) mediated cell necrosis processes, ferroptosis is also driven by unrestricted lipid peroxidation, mainly forming reactive oxygen species (ROS) that attack lipids, especially the polyunsaturated fatty acids (PUFAs) in cellular and organelle membranes [3]. Besides, although the interest from basic and clinical researchers in ferroptosis has increased in the past decade, molecular targets and regulatory mechanisms of ferroptosis remain plenty of unknowns [4].

Tumour cells’ obvious epigenome characteristics, such as DNA methylation, histone covalent modification, and chromatin remodelling, greatly affect their occurrence or development [5]. Increasing evidence indicates that epigenetic regulation is closely related to ferroptosis [6]. For instance, the bromodomain protein subfamily (BRDs) is one of the most important epigenetic transcriptional regulators in cancer cells, which is characterized by the representative bromodomain and the extra terminal (BET) domain [7]. Four family members of BRDs: BRD2, BRD3, BRD4 and BRDT, have been identified, and among them, the role of BRD4 in cancers is the most well studied [8]. BRD4 is widely recognised in the assembly of super-enhancers (SEs) and has been proven to promote the expression of oncogenes [9]. Studies have reported that inhibition of BRD4 blocked the communication between SEs and oncogenes’ target promoters, which is the most trusted mechanism for BRD4 inhibitors, such as (+)-JQ1 and the likes [10]. Based on this function, BRD4 inhibition is considered a promising treatment for blood and solid malignancies [11, 12]. However, only a few studies have focused on BRD4 and ferroptosis, we still don’t know for sure what roles BRD4 might play in ferroptosis.

Since cancer cells usually show abnormally active levels of lipid metabolism to meet the needs of survival and proliferation, dysregulation of lipid metabolism has been identified as a significant metabolic alteration in cancer [13]. In addition, studies have shown that lipid metabolism has a significant effect on ferroptosis [14]. Several enzymes that are involved in regulating the synthesis and disassembly of PUFAs, such as the long chain fatty acid CoA ligase 4 (ACSL4), lysophospholipid acyltransferase 5 (LPCAT3), and polyunsaturated fatty acid lipoxygenase ALOX15 (ALOX15), have also been reported to affect ferroptosis sensitivity [15, 16]. However, much remains unclear about the regulatory mechanisms of lipid metabolism during ferroptosis.

Historically, ferroptosis was discovered and named by chance while screening for inhibitors that selectively kill RAS mutated cancer cells [3]. Inspired by this, 464 commercial inhibitors for 164 different targets were selected in this study to screen the targets that significantly affect ferroptosis induced by erastin or RSL3 (the two commonly used inducers of ferroptosis). Although inhibitors of BRD4 showed a strong antagonistic effect on erastin-induced ferroptosis, they did not appear to have the same significant effect on RSL3, nor did they significantly affect glutathione peroxidase 4 (GPX4). This study provides a new epigenetic regulatory mechanism of fatty acid metabolism controlled by BRD4, showing its marked impact on erastin-induced ferroptosis.

## Materials and methods

### Cell culture and treatment

HT1080 and CALU1 cells, purchased from Fenghui Biotechnology (Changsha, China), were cultured in high glucose DMEM (Gibco, Cat. NO. 11965-092) supplemented with 10% foetal bovine serum (Invitrogen, Cat. NO. 16000-044) at 37 °C, 5% CO_2_ in humidified air. Before plasmid transfection, cells were serum-starved with 0.5% FBS for 12 h.

### Cell viability assay

HT1080 or CALU1 were plated into 96- well plates, then inhibitors, erastin and RSL3 were added to the cells in desired concentrations and left to stand for 24 h. Subsequently, cell viability was detected using an MTT assay kit, following the manufacturer’s instructions (Merck, Cat. NO. 11465007001), after which absorbance was measured at 450 nm using a microplate reader (Bio-Rad Laboratories, Cat. NO. 1681135).

### Over expression and interference of BRD4

While the CDS of the BRD4 gene was introduced into the expression vector pcDNA3.1, shRNAs targeting the CDS of BRD4 were introduced into an interference vector hU6. Subsequently, the expression level of BRD4 in cells transfected with over-expressed or interference vectors was detected by real-time polymerase chain reaction (qRT-PCR) and western blotting. Sequences of shRNAs and qRT-PCR primers are listed in Table 1 and Table 2.

**Table 1:** Sequence for primers.

Pirmer	Sequence 5'-3'
BRD4 (for qPCR)	F: GTGGTGCACATCATCCAGTC R: CCGACTCTGAGGACGAGAAG
HADH (for qPCR)	F: AAAGAGGTTGGCCTCAGCAG R: ATCTCCGCAGGGTCATGTTC
ACSL1 (for qPCR)	F: CGGTGTCATCCCCTTACTCTG R: AAGGGGCAGGGTTCACTGTA
ACAA2 (for qPCR)	F: CCATGGCAATGACTGCAGAG R: GTAGCCAGCATCATTAGCAGC
GAPDH (for qPCR)	F: CTGACTTCAACAGCGACACC R: GTGGTCCAGGGGTCTTACTC
β-actin (for qPCR)	F: CCCTGGAGAAGAGCTACGAG R: CGTACAGGTCTTTGCGGATG
β-tubulin (for qPCR)	F: GAGGGGCATCTCTTGAGAAC R: CTGATGACTTCCCAGAACTGT
HADH (for ChIP)	F: GCTCAACGCTGGGACGTT R: GACTCTGGGGGCGGG
ACSL1 (for ChIP)	F: CCACAGAACTTGGGAGGTGC R: TTTCACTGGTTCCCGCTTGG
ACAA2 (for ChIP)	F: CCTACGCACTCAAAAGAAGCC R: GCCGATGATGACGAGACCA
TCONS_00008546 (for qPCR)	F: GCCTAAAGTTGCCTCCCTACC R: GCCTGTAATGGGCTAAAAGCT
TCONS_00008193 (for qPCR)	F: TTGGGGTAGAGGTGAGGGTG R: GCAGGTCTTTGGACAACGAAT
TCONS_00008694 (for qPCR)	F: GGCAGAGTGTAAATGAGATGGG R: CTGGCAGTAAGACAGGAAGGAC
TCONS_00008695 (for qPCR)	F: CCCACAGGCGAAGAAGGAA R: GGGGCAATCAAGGCACATC
mgU12-22/U4-8 (for qPCR)	F: TGAGCCCGCAGTATTTTCC R: TCATCGGCCAGGACTCACAG
TCONS_00026520 (for qPCR)	F: AGAACTCCCATCTCCACATCCT R: CACTCCACAAAGCCAACTGAA

**Table 2:** Sequence for shRNA, siRNA and sgRNA.

Targets	Sequence 5'-3'
BRD4 (shRNA)	CCAGAGTGATCTATTGTCAAT
BRD4 (siRNA)	GGAAAGAGGAAGUGGAAGATT UCUUCCACUUCCUCUUUCCTT
HMGB2 (siRNA)	GGAUGAAGAUGAAGAAUAATT UUAUUCUUCAUCUUCAUCCTT
HADH (sgRNA)	GTCTGTGCTGTGGACAACGG
ACSL1 (sgRNA)	GTTCCACTTTATGATACCCT
ACAA2 (sgRNA)	CTTGTCTGAATTTGCTGCCA

### qRT-PCR

Total RNA was isolated from tissue samples and cells using TRIzol Reagent (Invitrogen, Cat. NO. 15596-026), followed by reverse transcription using a cDNA reverse transcription kit (Takara, Cat. NO. RR037A). Subsequently, qRT-PCR was conducted using SYBR green PCR master mix (Takara, Cat. NO. RR430B). GAPDH and β-actin were used as an internal control for normalisation. Finally, the relative expression of target genes was determined using the 2^-ΔΔCT^ method. The primers used for qRT-PCR in this study are listed in Table 1.

### Western blotting

Total protein lysates from cells were separated by sodium dodecyl sulfate-polyacrylamide gel electrophoresis and transferred onto a polyvinylidene difluoride membrane (Millipore, Massachusetts, USA). Then, primary antibodies were diluted to a working concentration and incubated overnight at 4 °C. Subsequently, the corresponding horseradish peroxidase (HRP)-conjugated secondary antibodies were incubated at room temperature for one hour, after which signals were detected by chemiluminescence using a Chemidoc system (Bio-Rad, Munich, Germany). Data were finally analysed using ImageJ software V1.8.0 (National Institutes of Health, Maryland, USA). Antibodies used in western blotting are listed in Table 3.

**Table 3:** Antibody working information.

Antibody	Vendor	Catalogue no.	Working dilution
BRD4	Abcam	ab243862	1:500 (WB); 1:50 (ChIP)
GPX4	Abcam	ab125066	1:1000 (WB); 1:100 (IHC)
Catalase	CST	14097	1:1000 (WB)
SOD1	CST	2770	1:1000 (WB)
Thioredoxin	CST	2285	1:1000 (WB)
Ferroportin1	MCE	HY-100579	1:1000 (WB)
FTH1	CST	2998	1:1000 (WB)
xCT	Abcam	ab175186	1:1000 (WB)
COX5A	Proteintech	11448-1-AP	1:500 (WB)
SDHA	Proteintech	14865-1-AP	1:500 (WB)
MTCO1	Abcam	ab203912	1:500 (WB)
ATP5F1	Proteintech	15999-1-AP	1:500 (WB)
H3K27ac	CST	8173	1:50 (IP)
Pol II	Abcam	ab264350	1:50 (IP)
P300	CST	54062	1:50 (IP)
HADH	Abcam	ab110284	1:1000 (WB); 1:50 (IF)
ACSL1	Abcam	Ab189939	1:1000 (WB); 1:50 (IF)
ACAA1	Abcam	ab1289293	1:1000 (WB); 1:50 (IF)
HMGB2	Abcam	ab67282	1:10 (IP)
AMPK	CST	5831	1:1000 (WB)
p-AMPK	CST	2535	1:1000 (WB)
ACC	CST	3676	1:1000 (WB)
p-ACC	CST	11818	1:1000 (WB)
β-actin	CST	5125	1:2000 (WB)
GAPDH	CST	3683	1:2000 (WB)
β-tubulin	CST	2146	1:1000 (WB)
Goat Anti-Rabbit IgG H&L (HRP)	Abcam	ab6721	1:5000 (WB)
Goat Anti-Mouse IgG H&L (HRP)	Abcam	ab97023	1:5000 (WB)
Goat Anti-Mouse IgG H&L (Alexa Fluor® 488)	Abcam	ab150113	1:500 (IF)
Goat Anti-Rabbit IgG H&L (Alexa Fluor® 488)	Abcam	ab150077	1:500 (IF)

### Lipid ROS detection

First, 10 μmol/L boron-dipyrromethene C-11 probe (Millipore, Cat. No. MX5211) was added and incubated with cells in a 5% CO_2_ incubator at 37 °C for one hour. Then, fluorescence images were taken under an inverted fluorescence microscope (Leica, Cat. No. DM2000LED) to analyse intracellular lipid ROS.

### Determination of iron content (Prussian-blue staining)

The cell slides were fixed with fixative for 30 s, rinsed with distilled water, and drained with filter paper. The smear was stained at 37 °C for 60 min (Abcam, Cat. No. ab150674) by dripping or immersing the working solution, then thoroughly rinsed with distilled water for 5 min. The water was drained by filter paper, and counterstained with nuclear solid red solution for 1–2 min. The smear was rinsed with distilled water and examined by microscopy after drying. Staining Interpretation: nuclei appear red, cytoplasm pink, and iron bright blue.

### Construction and administration of pulmonary metastatic tumour mouse model

BALB/C nude mice (SJA laboratory animal CO. LTD, Hunan, China) were fed adaptively for seven days, after which they were injected with a CALU1 cell suspension (5 × 10^6^ per mouse) through the tail vein to construct a mouse lung metastatic tumour model. Then, tumour-forming mice were randomly assigned to receive tail vein administration at the period set by the protocol. The injection dose comprised (+)-JQ1 (4 mg/mL, 50 μL/time) and erastin (1.75 mg/mL, 50 μL/time). The body weight of the mice was finally measured and recorded every two days during the observation, followed by in vivo imaging of each mouse (PerkinElmer, Cat. No. IVIS Lumina LT Series III). After the observation period, mice were euthanised using a one-time intraperitoneal injection of 7 mg/100 g excess pentobarbital solution and lung tissue samples were stripped for subsequent testing. The Experimental animal Welfare ethical review consent of The Second Xiangya Hospital of Central South University (2022014) approved animal experiments.

### MDA assay

Mice lung tissues were ground into tissue homogenate and centrifuged (10,000 × g, 10 min) to collect supernatant. MDA content in all samples were then tested and calculated according to kit operasting instruction (Beyotime, Cat. No. S0131S).

### Histochemical staining

Fresh lung tissues of mice were fixed with 10% formalin (Servicebio, Wuhan, China), then made into paraffin sections at 5 μm. Subsequently, these sections were dewaxed, rehydrated and stained with haematoxylin and eosin (H&E) as previously described [17].

### Immunohistochemistry (IHC)

Paraffin sections were subjected to antigen retrieval. After blocking with 10% goat serum, slides were incubated overnight with GPX4 primary (Abcam, Cat. No. ab125066) at 4 °C and HRP-conjugated secondary (Abcam, Cat. No. ab205718) antibodies at room temperature for one hour. Signals produced were finally visualised using a mouse and rabbit-specific HRP/AEC IHC detection kit (Servicebio, Wuhan, China).

### Electron microscopy

Cell samples were encapsulated and fixed in 1% agar. After dehydration, embedding and curing were conducted, followed by the preparation of ultrathin slides at 70 nm and staining with 2% uranium acetate and lead citrate. Finally, the images were taken using transmission electron microscopy (Hitachi, Cat. No. HT7800).

### Mito ROS detection

Cell precipitates were prepared, after which a pre-prepared MitoSOX™ Red fluorescent probe (Life, Cat. No. M36008) was added to the precipitates and incubated at 37 °C for 30 min away from light. After PBS resuspension, the cells were filtered with a 400 mesh sieve and detected using flow cytometry (CytoFlex, Cat. No. B53000).

### Oxygen consumption rate detection

A cell energy metabolism analyser (Agilent, Cat. No.Seahorse XFe96 Analyzser) was used to measure the oxygen consumption rate of cells in 96-well plates as previously described [18]. Results were finally analysed using the *Wave software 2.4.0* that was provided with the instrument.

### ATP assay

The ATP quantitative assay was implemented following the colorimetric/fluorometric method. An ATP assay kit (Abcam, Cat. No. ab83355) that relies on the phosphorylation of glycerol to generate a product that is easily quantified colorimetrically (ODmax = 570 nm) or fluorometrically (Ex/Em = 535/587 nm) was used. Briefly, cell lysate samples (deproteinised) and standards were added to wells with a reaction mix and incubated for 30 min at room temperature. Signals were subsequently detected using a microplate reader (Thermo Scientific, Cat. No. Multiskan Spectrum).

### Mitochondrial membrane potential detection

The JC-1 working solution was prepared as required. Then, cells were rinsed with PBS 1–2 times and detected according to the operation steps of the product instructions (MCE, Cat. No. Hy-15534). Finally, fluorescence signals were recorded under a fluorescence microscope (Leica, Cat. No. DM2000LED).

### ChIP-Seq and ChIP-qRT-PCR assays

Cell samples were lysed and ultrasonically broken to produce 200–500 bp chromatin fragments. Then, the fragments were precipitated following the kit manufacturer’s instructions using the BRD4 ChIP grade antibody (Abcam, Cat. No. Ab243862) (CST, Cat. No.9003). Genomic products precipitated by the BRD4-ChIP antibody were subsequently sent to Novogene Bioinformatics Technology Co. Ltd (Beijing, China) for genomic sequencing and bioinformatics analysis. However, qRT-PCR was conducted to amplify the promoter ACAA2, HADH, and ACSL1 fragments in ChIP products. Obtained data were finally displayed in percentage of the Input. The qRT-PCR primer sequences used for this analysis are listed in Table 1.

### Dual-luciferase assay

The following plasmids were co-transfected into 293 T cells with three to five parallel repeat holes: psicheck2 & pcDNA3.1-NC, psicheck2 & pcDNA3.1-BRD4, psicheck2 HADH/ACSL1/ACAA2 -promoter (wide or mutation) pcDNA3.1 & pcDNA3.1-NC, and psicheck2 HADH/ACSL1/ACAA2-promoter (wide or mutation) & pcDNA3.1-BRD4. Cells were then lysed and centrifuged, after which the supernatant was collected and placed on a multifunctional enzyme reader (BLT, Cat. No. Lux-t020) to determine the luciferase activity of the sea cucumber/firefly. The specific steps followed were conducted according to the kit’s operasting instructions (Promega, Cat. No. E1910).

### Co-IP assay

First, the fusion expression vectors pcDNA3.1-BRD4 and pcDNA3.1-HMGB2 were constructed and transfected into CALU1 cells. IP grade antibodies of labelled proteins were used for the immunoprecipitation process. Then, the contents of target proteins in the precipitated products were detected by Western blotting. Antibodies used in co-IP and western blotting are listed in Table 3.

### Yeast one-hybrid system

The bait recombinant plasmid, pAbAi- HADH/ACSL1/ACAA2 and the prey recombinant plasmid, pGADT7-BRD4, were constructed and transferred into engineering yeast. When the fusion expression vector of the transcription factor (BRD4) was transferred into the yeast, it activated the Pmin promoter. However, the reporter gene expression was promoted after binding with the target cing-acting element (HADH/ACSL1/ACAA2 promoter fragment). Results were interpreted as follows: for yeast strains containing the bait promoter, pAbAi- HADH/ACSL1/ACAA2, yeast growing on the SD/-Ura plate means that bait vectors have been successfully transferred into the host bacteria and have no toxicity; yeast growing on the SD/-Ura/AbA (100 ng/mL) plate but could not grow on the SD/-Ura /AbA (200 ng/mL or 300 ng/mL) plates means the lowest AbA concentration that could inhibit reporter genes was 200 ng/mL; yeast growing on the SD/-Leu plate means that the prey recombinant plasmid, pGADT7-BRD4, has successfully been transferred into the host bacteria and has no toxicity and yeast growing on the SD/-Leu /+AbA (250 ng/mL) plate means that pAbAi- HADH/ACSL1/ACAA2 and pGADT7- BRD4 could interact and activate the reporter gene expression of host bacteria.

### Yeast two-hybrid system

Briefly, the prey vector pGADT7- BRD4 containing different BRD4 domains and a bait vector pGBKT7- HMGB2 were constructed and transferred into yeast. If the yeast could grow and turn blue on the SD/-Trp/-Leu/-His/-Ade plate, no self-activation happened, indicating an interaction between BRD4 and HMGB2. Meanwhile, the prey and bait identities of HMGB2 and BRD4 were changed to verify their binding relationships again.

### The co-localization of of HADH, ASCL1, ACAA2 and the mitochondria

The slivers of cells were doubly stained using immunofluorescence (IF) and a mitochondrial tracer dye Mito Tracker (Beyotime, Cat. No. C1035). Then, the slivers were scanned for each channel through whole slide imaging (3D-HISTECH, Cat. No. P250 FLASH) and merged for co-localization analysis. The antibody information is listed in Table 3.

### Knockout of HADH, ACSL1, and ACAA2

gRNAs targeting HADH, ACSL1, and ACAA2 were designed and synthesized on the pSpCas9 (BB)-2A-Puro (PX459) vector. Then, Sanger’s sequencing examined the efficiency of gene knockout by sgRNAs. The sgRNA sequences used are listed in Table 2.

### PUFA content assay

Sci-tech Innovation Co., LTD. (Shan Dong, China) provided targeted fatty acid detection and analysis in this study. In brief, this assay comprised three main steps: extraction of lipid samples from cells, saponification and methyl esterification, then the absolute determination of target fatty acid content in samples by gas chromatography. The data acquisition instrument system (Agilent, Cat. No.7890 A) was finally used to collect and analyse the GC data.

### Statistical analysis

Results are presented as mean ± SD unless mentioned otherwise. While two-tailed unpaired Student’s *t*-test determined the statistical significance for two-group comparison, one-way ANOVA with Tukey or Dunnett was conducted for multiple group comparison using GraphPad Prism 8 (GraphPad software). *P* < 0.05 and |Log2 FC | > 1 were considered significant. Significance in all figures was indicated as follows: *P < 0.05, **P < 0.01, ***P < 0.001, N.S.: not significant.

## Results

### BRD4 inhibition limits erastin-induced ferroptosis in vitro and in vivo

**Fig. 1:**
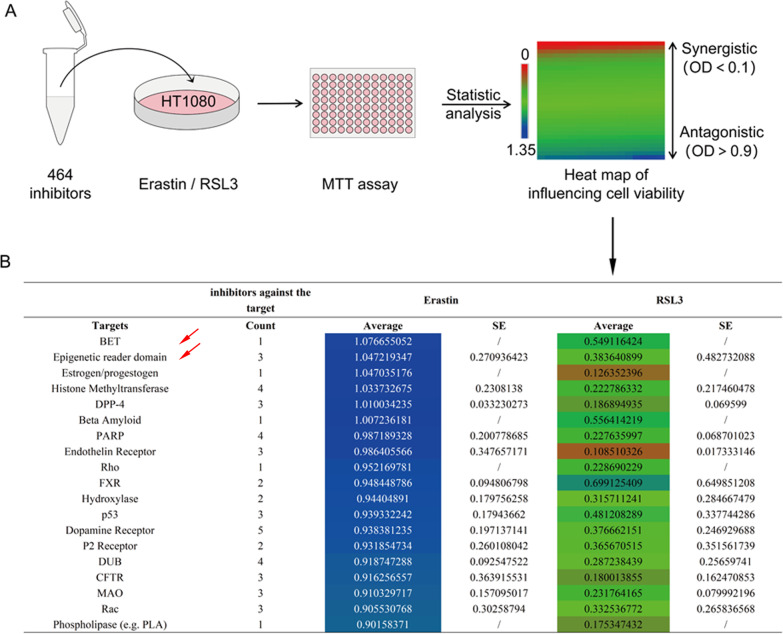
Inhibition of BRD4 significantly inhibited erastin-induced ferroptosis in vitro. **A** Flowchart showing the MTT screening and statistical analysis process. **B** List of the targets of which inhibitors significantly antagonised the effects on erastin-induced ferroptosis. In **B**, the mean OD values and the standard error (SE) were calculated from the separate OD values of the cells treated with similar target inhibitors and ferroptosis inducers: erastin or RSL3 (SE was absent if there was only one inhibitor of the target). OD = 0.5 is the dividing line, the reference OD value of cells treated with erastin and fer-1 (a ferroptosis inhibitor). Among these inhibitors, while 0.1 < OD < 0.9 (green background) indicated no significant effect of inhibitors on ferroptosis, OD ≤ 0.1 (yellow background) and OD ≥ 0.9 (blue background) were significantly promoted or antagonised ferroptosis. Four inhibitors were used for the BET and epigenetic reader domain: OTX015, PFI-1 (PF-6405761), I-BET151 and (+)-JQ1.

All commercial inhibitors (purchased from *Selleck Chemicals*) were prepared into a working solution (10 μM), then added to HT1080 (a ferroptosis sensitive cell strain) and combined with erastin or RSL3 at IC50 concentrations. Inhibitors that significantly antagonised erastin-induced ferroptosis (abbreviated as erastin-ferrop, unless otherwise specified) are presented. Among them, 4 BRD4 inhibitors: OTX015, PFI-1 (PF-6405761), I-BET151 and (+)-JQ1, showed the greatest average antagonism (Fig. 1A, B). The original database for this screening work have been uploaded in a public repository.

**Fig. 2:**
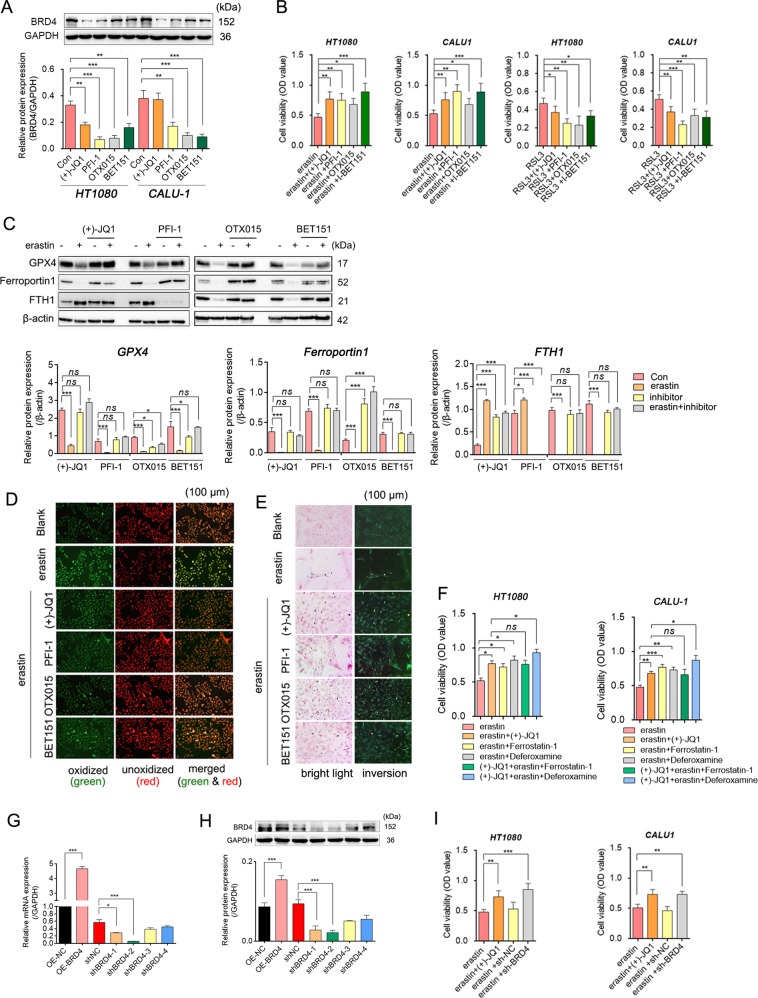
Inhibition of BRD4 suppress erastin-induced ferroptosis in vitro. **A** Relative BRD4 protein expression levels detected by western blotting (*n* = 3, Mean ± SD, Tukey–Kramer test of one-way ANOVA). **B** Cell viability analysis through the MTT assay (*n* = 5, Mean ± SD, Dunnett’s test of one-way ANOVA). **C** Relative protein expression levels tested by western blotting (*n* = 3, Mean ± SD, Tukey–Kramer test of one-way ANOVA). **D** Intracellular lipid ROS of each group, determined by the boron-dipyrromethene C-11 probe. Green fluorescence reflects the total number of cells, red fluorescence indicates lipid ROS and the merged image represents the distribution intensities of lipid ROS in cells. **E** Intracellular Fe^2+^ content tested by Prussian-blue staining. **F** and **I** Cell viability analysis through the MTT assay (*n* = 5, Mean ± SD, Dunnett’s test of one-way ANOVA). Relative mRNA and protein expression levels of BRD4 detected by qRT-PCR (**G**) and western blotting (**H**) (*n* = 3, Mean ± SD, Tukey–Kramer test of one-way ANOVA).

Inhibitors that targeting BRD4 showed good inhibitory effect on BRD4 protein level in HT1080 and CALU1 cells (Fig. 2A and Fig. S1). At the same time, they also significantly increased the cell viability under erastin treatment, but had no effect on RSL3-induced ferroptosis (Fig. 2B). To explore how BRD4 inhibitors affect ferroptosis, we assessed changes in lipid ROS and intracellular Fe^2+^content in the presence of inhibitors and erastin alone or in combination. Significantly reduced lipid ROS accumulation, as well as no significant changes in Fe^2+^ content or iron transporter (ferroportin1, FTH1) expression were observed in the combined treatment group than in the control group, although erastin alone had a classic effect on both (Fig. 2C–E and Fig. S2). Further, deferoxamine (a Fe^2+^ chelator) promoted cell activity in the presence of (+)-JQ1 towards erastin-ferrop significantly, but ferrostatin-1, a lipid ROS scavenger, did not (Fig. 2F). A plausible explanation for this is that BRD4 inhibition and Fe^2+^ neutralization are relatively independent of each other in their anti-ferroptosis effects, but overlap with lipid ROS scavenging. In addition to specific inhibitors, shRNA that targeting the coding sequence (CDS) of BRD4 was used to re-evaluate the effect of BRD4 expression on erastin-ferrop, and resultes showed that interfering with BRD4 expression also protected cells from erastin-ferrop (Fig. 2G–I and Fig. S3), suggesting that BRD4 is necessary for erastin-ferrop.

It’s worth noting that the protein expression of GPX4, whose decline was considered one of the signature events of ferroptosis [18, 19], did not change significantly when BRD4 inhibitor was treated alone or in combination with erastin, but decreased significantly when erastin was treated alone. Similarly, the antioxidant factors (catalase, SOD1, thioredoxin), iron transport factors (ferroportin1, FTH1), system xc- (xCT), and autophagy markers (LC3 and P62) responded positively to erastin but did not respond to BRD4 inhibitors. Moreover, their response to erastin was terminated after treatment with the inhibitor (Fig. S2A and Fig. S4), appearing that the antagonism to erastin-ferrop of BRD4 inhibition may be independent of oxidative stress, iron transport, cysteine transport and autophagy, but concerned with the lipid ROS. Another result that supports this hypothesis is the *Ingenuity Pathway Analysis* (IPA) of genes with significantly reduced BRD4 binding degree in their promoter regions after BRD4 inhibition by (+)-JQ1 treatment, showing that while the inhibition of BRD4 was not associated with iron storage, it was linked to lipid peroxidation and ROS accumulation (Fig. S2B).

**Fig. 3:**
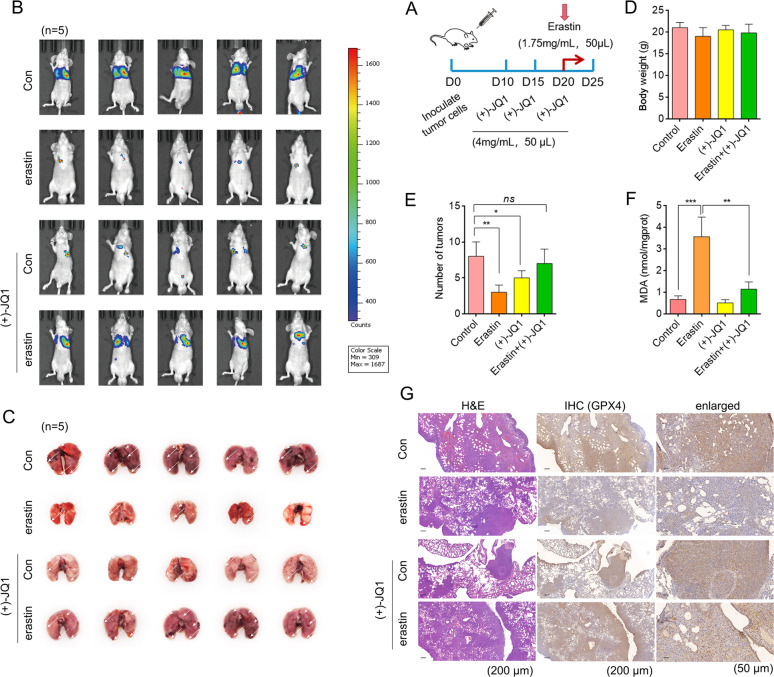
BRD4 inhibitors suppress erastin-induced ferroptosis in vivo. **A** Flowchart guide for the animal experiments. **B** In vivo imaging of mice with caudal vein lung metastases in each group. **C** Lung tissue from individual mice. **D** Animal weights. **E** Pulmonary nodule counts. **F** MDA contents. **G** H&E staining and IHC detection of lung tissue samples. (*n* = 5, Mean ± SD, Dunnett’s test of one-way ANOVA, *P < 0.05; **P < 0.01; ***P < 0.005; N.S.: no significance).

Next, nude mice were used to establish in vivo tumour models of lung metastases. The mice were treated with erastin and (+)-JQ1 alone or combined (Fig. 3A). Pictures from the in vivo imaging system showed that although both (+)-JQ1 and erastin had significant anti-tumour effects with fewer tumour nudes in the lungs, poor treatment occurred in the combined group (Fig. 3B–E). In addition, erastin significantly induced model driven architecture (MDA) levels (Fig. 3F) and depleting GPX4 contents (Fig. 3G) in lung tissues, but not in combination with (+)-JQ1.

Overall, these data support that the inhibition of BRD4 significantly protects cells from erastin-ferrop both in vitro and in vivo.

### Inhibition of BRD4 weakened mitochondrial function

Studies have reported that the excessive accumulation of lipid ROS that leads to ferroptosis is mainly formed by unrestricted mitochondrial ROS (mito ROS), attacking PUFAs of cellular or organelle membranes [3]. Therefore, since mitochondria’s electron transport chain (ETC) is the main place where mito ROS is produced [20], it is a priority to examine mitochondrial metabolic flux before and after BRD4 inhibition.

**Fig. 4:**
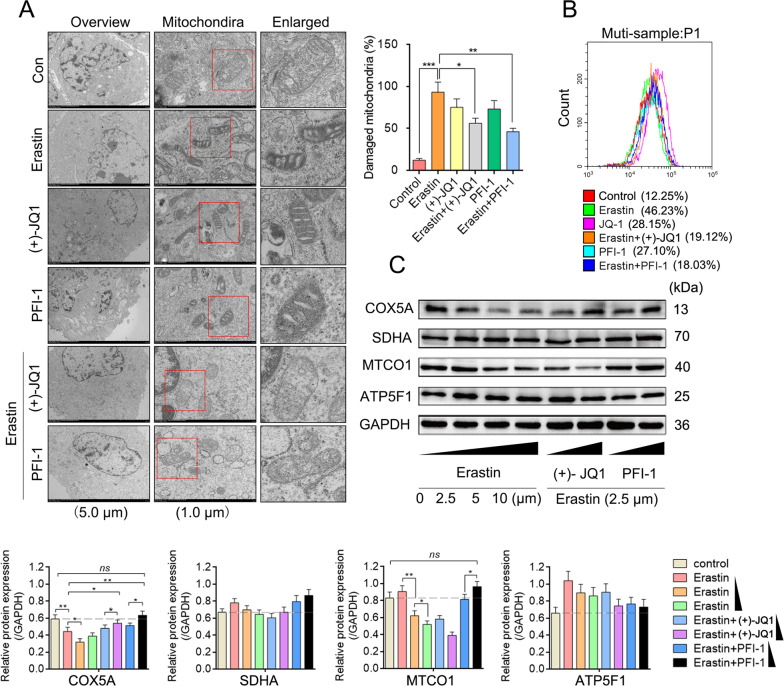
Inhibition of BRD4 significantly protected respiratory chain complex III from mitochondrial ROS. **A** Electron microscopic images of the mitochondria and the percentage of the damaged mitochondria. **B** Determination results of the mito ROS content using a mito SOX probe. **C** Western blotting showing the respiratory chain complex’s protein SDHA/COX5A/MTCO1/ATP5F1 levels (*n* = 3, mean ± SD, Tukey–Kramer test of one-way ANOVA, *P < 0.05; **P < 0.01; ***P < 0.005; N.S.: not significant).

In the erastin group, the mitochondria exhibited typical ferroptosis features (Fig. 4A) with the increased mito ROS (Fig. 4B). Notably, we observed that the protein expression levels of cytochrome C oxidase subunit 5 A (COX5A) and cytochrome C oxidase subunit 1 (MTCO1) (important components of respiratory chain complexes III of the mitochondria) markedly reduced when erastin was applied. However, other complex components, such as the succinate dehydrogenase [ubiquinone] flavoprotein subunit (SDHA, involved in complex II) and the ATP synthase F(0) complex subunit B1 (ATP5F1, involved in complex V), showed no significant changes (Fig. 4C and Fig. S6). These results prove that erastin could trigger ferroptosis by destroying complex III of the ETC and elevating mito ROS.

In the BRD4 inhibited group, while smaller mitochondrial volumes and less medial crests were observed, we detected relatively clear structures in the mitochondrial bilayer membranes (Fig. 4A). But the expression levels of COX5 A and MTCO1, as well as the total mito ROS was much more closer to the control group in the erastin-ferrop groups when BRD4 inhibitors (PFI-1 and (+)-JQ1) were applied (Fig. 4B, C). In view of the complexes III is the major source of mito ROS production [21], and the excessive destruction of respiratory chain complexes leads to elevated mito ROS levels [22], these findings suggest that inhibiting BRD4 helps protect the ETC from erastin and avoids excessive ROS production.

**Fig. 5:**
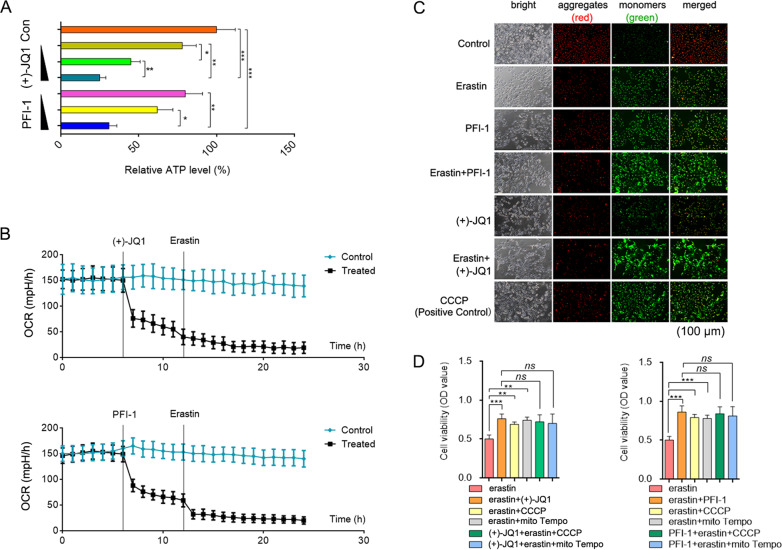
Inhibition of BRD4 decreased oxidative catabolism of cells. **A** ATP content quantified colorimetrically (ODmax = 570 nm) or fluorometric (Ex/Em = 535/587 nm) methods (*n* = 3, Mean ± SD, Tukey–Kramer test of one-way ANOVA, *P < 0.05; **P < 0.01; ***P < 0.005). **B** Oxygen consumption rate measured by the cell energy metabolism analyzer (*n* = 5). **C** Mitochondrial membrane potential tested using the Jc-1 method. **D** Cell viability by the MTT assay (*n* = 5, Mean ± SD, Tukey–Kramer test of one-way ANOVA, *P < 0.05; **P < 0.01; ***P < 0.005; N.S.: not significant).

Meanwhile, we measured the oxidative catabolism of the cells before and after BRD4 inhibition. Compared with the erastin-processed groups, a much lower ATP production (Fig. 5A), oxygen consumption rate (Fig. 5B), and mitochondrial membrane potential (Fig. 5C) were detected when BRD4 was inhibited by its inhibitors, (+)-JQ1 and PFI-1. Moreover, while (+)-JQ1 and PFI-1 improved cell viability during erastin treatment, the proton uncoupling agent (CCCP) and mito ROS scavenger (Mito Tempo) had no significant effect on erastin-ferrop after BRD4 inhibition (Fig. 5D). This is proposed to be because the inhibition of BRD4 itself can inhibit mitochondrial function that makes CCCP or Mito Tempo has little effect.

Together, these results demonstrate that inhibition of BRD4 significantly reduces mitochondrial metabolic flux and protects respiratory chain complexes III from mito ROS under erastin processing conditions.

### BRD4 enhances the transcriptional activity of fatty acid metabolism genes HADH, ACSL1 and ACAA2

**Fig. 6:**
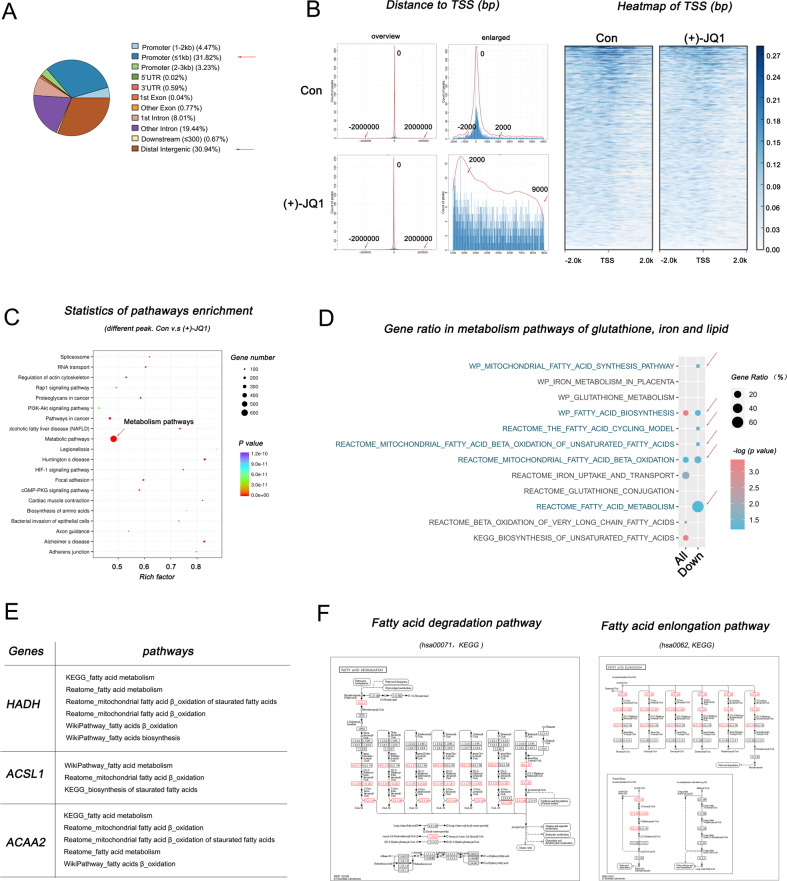
HADH, ACSL1, and ACAA2 as fatty acid metabolism genes that regulated by BRD4. **A** Pie chart of the distribution of BRD4-bound chromatin fragments. **B** Analysis of BRD4 binding region distribution in chromatin. **C** Gene enrichment pathways corresponding to BRD4-bound chromatin fragments. **D** Gene enrichment analysis results of the GSH synthesis, iron metabolism, and lipid metabolism pathways from *KEGG, Reactome*, and *WikiPathway* database, respectively. **E** Genes involved in fatty acid metabolism pathways reflected in all (*KEGG, Reactome* and *WikiPathway*) databases. **F** Maps of HADH, ACSL1, and ACAA2 from *KEGG* pathway database. In **C** and **D**, the scatterplot is a graphical grouping display method based on the results of the gene enrichment analysis. *GeneRatio* refers to the ratio between the number of genes enriched in the term and the number of annotated genes. P. adjust refers to the *p*-value of each term calculated using the Fisher test (the probability of hypothesis test). The smaller the *p*-value, the greater the significance and the lower the misjudgment rate. In **F**, red boxes represent the metabolic steps involved in HADH, ACSL1 and ACAA2.

Subsequently, we explored the molecular mechanism of BRD4 affecting erastin-ferrop, using chromatin immunoprecipitation (ChIP) to obtain BRD4-binding chromatin fragments before and after BRD4 inhibition by (+)-JQ1 treatment. As can be seen that BRD4 primarily binds around the transcription start sites (TSS) of individual genes (−1000bp ~ 1000 bp, 31.82%) and distal intergenic (30.94%) (Fig. 6A, B), which is consistent with its enhancer assembly identity. Statistics of pathway enrichment showed that most of the genes that BRD4 binding degree were significantly reduced were enriched in metabolic pathways (Fig. 6C). Further, the enrichment in glutathione (GSH) metabolism, Fe^2+^ metabolism, and lipid metabolism pathways were analyzed separately because of their strong association with ferroptosis. Results showed that the fatty acids metabolism pathways were noteworthy (Fig. 6D), and there are three genes include Hydroxyacyl-coenzyme A dehydrogenase (HADH), Long-chain-fatty-acid-CoA ligase 1 (ACSL1), and 3-ketoacyl-CoA thiolase(ACAA2) involved in fatty acid metabolism pathways from all of *KEGG*, *Reactome*, and *WikiPathway* database (Fig. 6E). It was coloured which HADH, ACSL1, and ACAA2 were participated in in *KEGG* maps has00071 (fatty acid degradation) and hsa0062 (fatty acid enlongation) (Fig. 6F). The original information of BRD4-bound DNA sequences through ChIP/sequencing have been uploaded in a public repository.

**Fig. 7:**
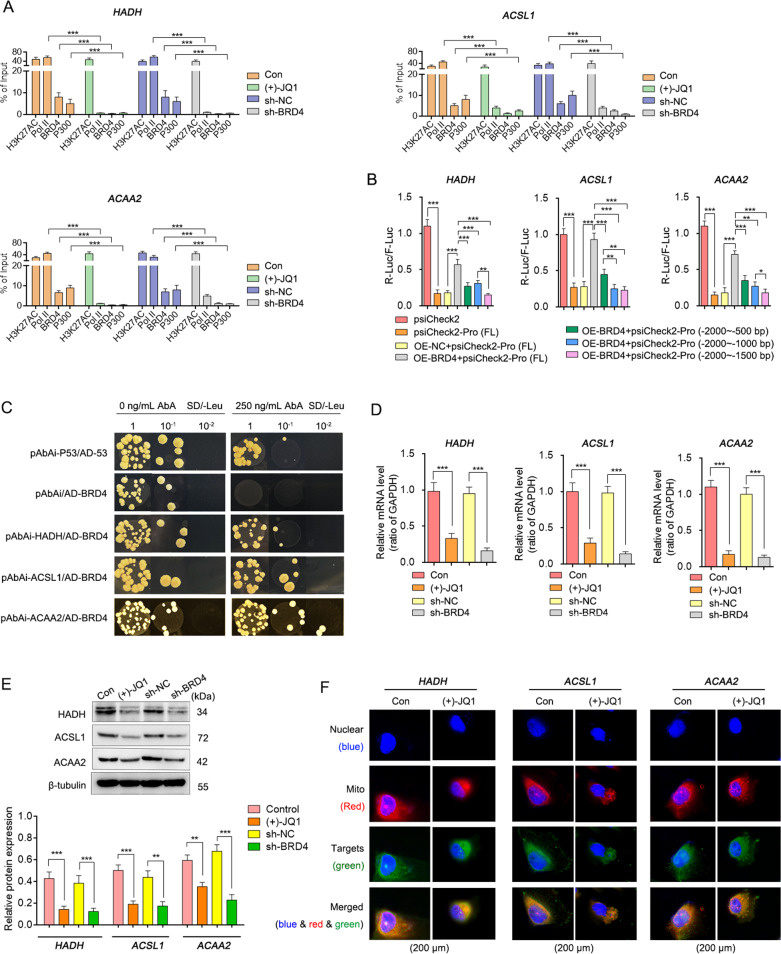
BRD4 promotes the transcription levels of HADH, ACSL1 and ACAA2 genes. **A** The enrichment of BRD4, H3K27ac, P300, and Pol II in the promoter region of HADH, ACSL1, and ACAA2 via the ChIP/ qRT-PCR assay. Transcription activity of BRD4 on HADH, ACSL1, and ACAA2 promoters regions via dual-luciferase assay (**B**) and yeast one-hybrid system **C**. (*n* = 3, Mean±SD, one-way ANOVA, *P < 0.05; **P < 0.01; ***P < 0.005; N.S.: not significant). The mRNA (**D**) and protein (**E**) expression levels of HADH, ACSL1, and ACAA2 detected by qRT-PCR and Western blotting (*n* = 3, Mean ± SD, one-way ANOVA, *P < 0.05; **P < 0.01; ***P < 0.005; N.S.: no significant). **F** Co-localization analysis between HADH/ ACSL1/ ACAA2 and the mitochondria.

There are abundant predicted BRD4 binding sites in HADH, ACSL1 and ACAA2 promoter regions, especially within 500 bp away from TSS. Given that BRD4, histone H3 (acetyl K27), histone acetyl-transferase p300 (P300), as well as RNA polymerase II (Pol II) are core components of SEs, their binding efficiencies in HADH, ACSL1 and ACAA2 promoter regions were detected before and after BRD4 inhibition. Results showed that except for H3K27ac, the binding degrees of BRD4, P300, and Pol II were significantly weakened by (+)-JQ1 or RNA interference of BRD4 (Fig. 7A). Further, BRD4 was proved to promote the transcriptional activities of these genes through the dual-luciferase assay and the yeast one-hybrid system (Fig. 7B, C). Alternatively, both mRNA and protein levels of HADH, ACSL1 and ACAA2 were detected and found to have significantly decreased after BRD4 inhibition (Fig. 7D, E). In addition, they were also observed to be basically co-located with mitochondria (Fig. 7F).

**Fig. 8:**
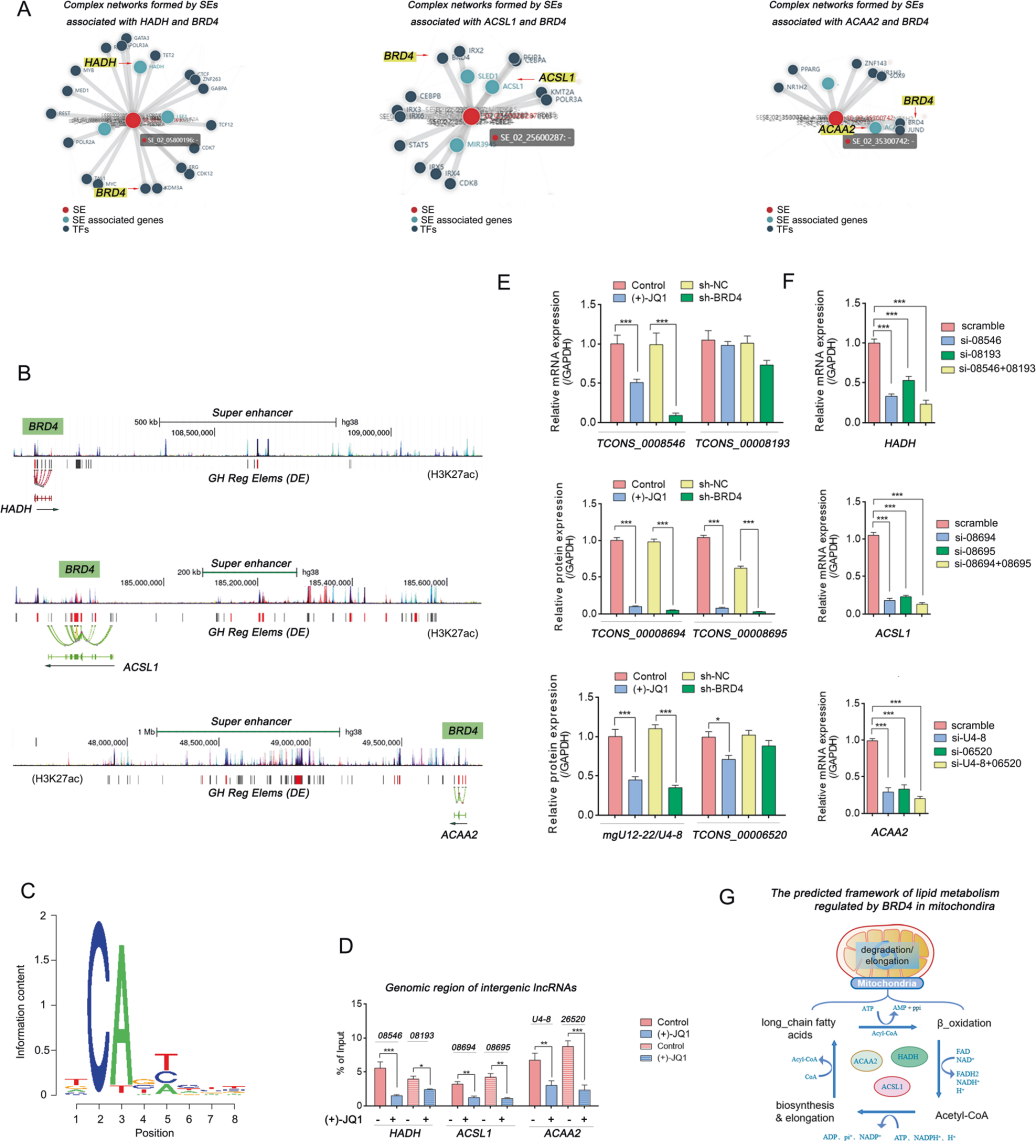
SEs information of BRD4 binding in HADH, ACSL1, and ACAA2. **A** Networks of SEs that bind to BRD4 and associated with HADH, ACSL1, and ACAA2. **B** Genomic locations of SEs. **C** Motif of BRD4 binding sites. **D** The enrichment of BRD4 in the genomic regions of intergenic lncRNAs that near the target mRNA genes via the ChIP/ qRT-PCR assay. **E**, **F** Relative mRNA levels detected by qRT-PCR (*n* = 3, Mean ± SD, one-way ANOVA, *P < 0.05; **P < 0.01; ***P < 0.005). **G** The predicted framework of fatty acid metabolism regulated by BRD4 in mitochondria.

Next, the SEs information searched from *SEanalysis* and *SEdb* databases that associated with HADH, ACSL1 and ACAA2 were supplemented (Fig. 8A). The locations of these SEs in the genomes were displayed (Fig. 8B) and the potential binding motif of BRD4 were revealed and verified by the ChIP-assay (Fig. 8C, D). As the expression level of lncRNA transcribed from super-enhancer regions (SE-lncRNA) is one of the evaluation criteria for the active SEs regulating target genes [23], the changes of these lncRNA transcripts were detected before and after BRD4 inhibition. Results showed that the intergenic lncRNAs, including TCONS_00008546/TCONS_00008193 (HADH), MGU12-22 /U4-8/ TCONS_00026520 (ACAA2), and TCONS_00008694/TCONS_00008695 (ACSL1), were significantly decreased while BRD4 was inhibited (Fig. 8E). Moreover, when these SE-lncRNAs were interfered by siRNAs, the mRNA levels of HADH, ACSL1 and ACAA2 were significantly reduced (Fig. 8F), indicating that inhibition of BRD4 may inhibit the transcriptional activity of target genes by decreasing the activity of related SEs. In terms of the biological function of HADH, ACSL1 and ACAA2, we mapped the predicted lipid metabolic framework which may controlled by BRD4 (Fig. 8G).

### BRD4 and enhancer binding factor HMGB2 jointly promote the transcription of HADH, ACSL1 and ACAA2

**Fig. 9:**
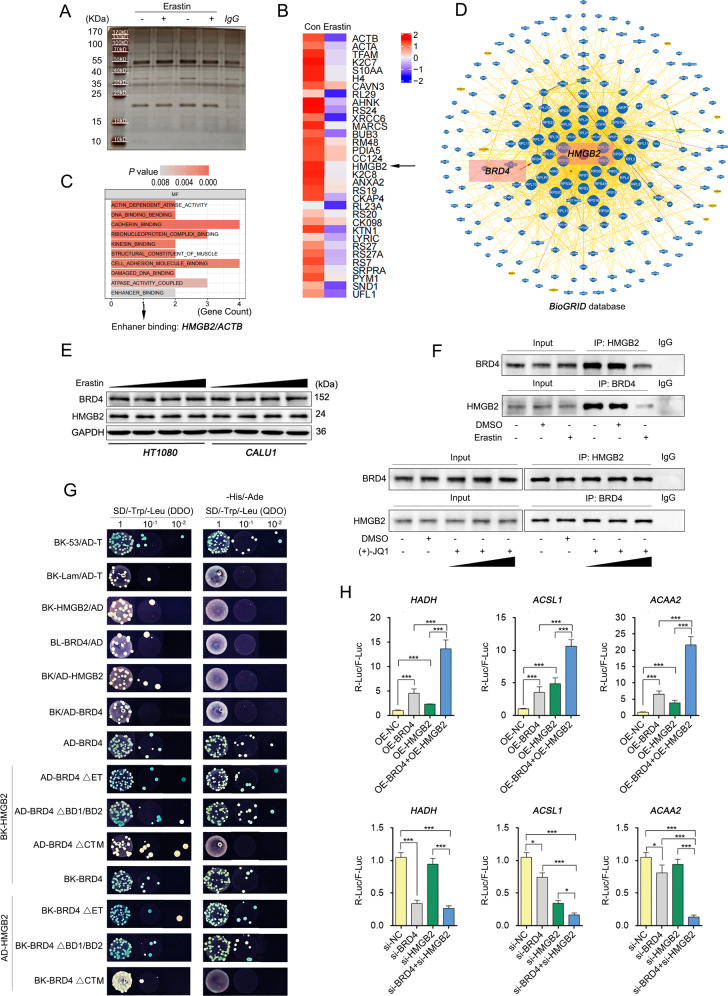
BRD4 binds HMGB2 to promote transcription levels of HADH, ACSL1, and ACAA2. **A** The co-IP silver staining diagram. **B** Heat map showing ignificantly down-regulated binding proteins. **C** Molecular function (MF) of significantly down-regulated binding proteins through GO enrichment analysis. **D** Network diagram showing BRD4 and HMGB2 from the *BioGRID* database. **E** Relative protein expression levels detected by western blotting. **F** Co-IP detection of the BRD4 and HMGB2 combination. **G** Yeast hybrid system to verify the binding domain of BRD4 (BD1, BD2, ET, and CTD domains) to HMGB2. **H** Dual-luciferase results showing the effects of BRD4 and HMGB2 over-expression (or down-regulation) on HADH, ACSL1, and ACAA2 promoter activity. (*n* = 3, Mean ± SD, one-way ANOVA, *P < 0.05; **P < 0.01; ***P < 0.005; N.S.: no significant).

BRD4 has four domains, of which the BD1, BD2 and ET domains can recognize and bind to acetylated chromatin regions, However, the CTM domains recruit transcription factors and form SEs to enhance the transcriptional activity of target genes [8]. In this study, BRD4-binding proteins were obtained through co-immunoprecipitation (Co-IP) and mass spectrum (MS) to identify the co-regulators with BRD4 during transcription enhancement (Fig. 9A). Proteins that bind to BRD4 significantly down-regulated after erastin treatment were classified and analyzed by gene ontology (GO). Results showed that HMGB2 (with enhancer binding capability) acted with BRD4 (Fig. 9B, C). Additionally, the *BioGRID* database supported this relationship between them (Fig. 9D). It was confirmed that their protein content of BRD4 and HMGB2 was not affected by erastin (Fig. 9E and Fig. S7), but the binding relationship between them was significantly weakened after erastin treatment, while (+)-JQ1 has no effect on this (Fig. 9F and Fig. S8). Moreover, the yeast two-hybrid system further demonstrated that the CTM domain of BRD4 acted on HMGB2, but not on the BD1, BD2 and ET domains (Fig. 9F). This result is proposed to be because the inhibitory mechanism of (+)-JQ1 is to bind to the BET domain of BRD4 competitively, resulting in proteins that bind to other domains being unaffected. Next, the dual- luciferase assay showed that co-over expression of BRD4 and HMGB2 significantly improved the transcriptonal activity of HADH, ACSL1 and ACAA2 than that of BRD4 or HMGB2 over-expression alone, and knocking down BRD4 or HMGB2 by siRNAs reduced the transcriptional activity of these genes. Besides, knocking down both of them resulted in a more significant inhibition (Fig. 9G). This finding suggests that BRD4 has a stronger transcriptional activation effect on HADH, ACSL1 and ACAA2 promoters when HMGB2 is involved. In this section, the BRD4-binding peptides identified by Co-IP/MS were uploaded in a public repository.

### The regulatory axis of lipid metabolism controlled by BRD4 enhances fatty acid metabolism and cell sensitivity to ferroptosis

**Fig. 10:**
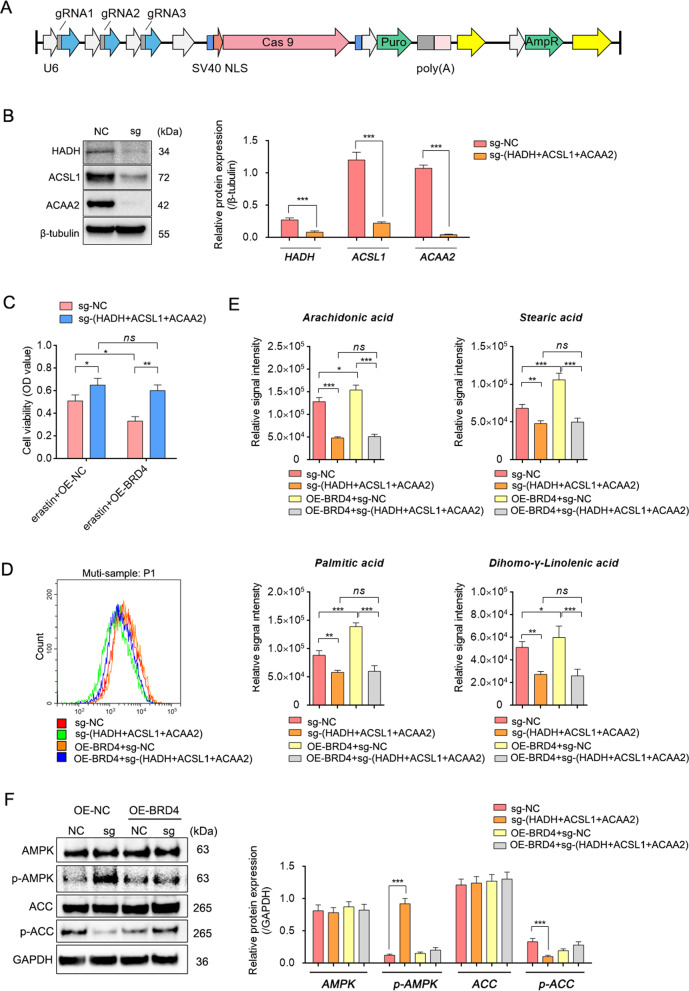
Inhibition of BRD4 results in reduced PUFAs. **A** Schematic diagram of vector pSpCas9(BB)-2A-Puro (PX459) with gRNAs target HADH, ACSL1, and ACAA2. **B** Relative protein levels detected by western blotting. (*n* = 3, Mean ± SD, Tukey–Kramer test of one-way ANOVA, *P < 0.05; **P < 0.01; ***P < 0.005). **C** MTT detected the cell viability of each group (*n* = 5, Mean ± SD, one-way ANOVA, *P < 0.05; **P < 0.01; ***P < 0.005; N.S.: no significant). **D** Lipid ROS detected by the BODIPY C-11 probe. **E** PUFA (stearic acid, palmitic acid, arachidonic acid, double high -γ -linoleic acid) contents in cell samples of each group were detected by targeting fatty acid metabolism (*n* = 3, Mean ± SD, one-way ANOVA, *P < 0.05; **P < 0.01; ***P < 0.005; N.S.: no significant). **F** *P*rotein expression levels detected by western blotting (*n* = 3, Mean ± SD, one-way ANOVA, *P < 0.05; **P < 0.01; ***P < 0.005; N.S.: no significant).

Plasmid with sgRNAs against HADH, ACSL1 and ACAA2 were designed (Fig. 10A, B and Fig. S9) and transfected into the BRD4 over-expressed cells with or without erastin treatment. The cell viability test and lipid ROS investigations revealed that the over-expression of BRD4 made cells more sensitive to erastin-ferrop but insensitive in the sgRNA groups even though BRD4 was over-expressed (Fig. 10C, D). Furthermore, the intracellular long-chain PUFA contents, such as stearic acid, palmitic acid, arachidonic acid and double high -γ -linoleic acid were increased in BRD4 up-regulated cells, but reduced significantly in the sgRNA groups with or without BRD4 over-expression (Fig. 10E). Additionally, the activity of the (AMP)-activated protein kinase (AMPK)/acetyl-CoA carboxylase (ACC) signalling pathway, which inhibits intracellular lipid synthesis when cells suffer from energy stress with low ATP, was tested. Results proved that when BRD4 was over-expressed, the AMPK activity was repressed and then increased ACC, which was favourable for lipid synthesis. However, the inhibition of AMPK/ACC pathway by BRD4 depends to some extent on the presence of these genes (Fig. 10F and Fig. S10). These findings suggest that not only did the inhibition of BRD4 cause ferroptosis insensitivity, but the inhibition of its regulated downstream genes (HAHD, ACSL1 and ACAA2) also had an effect, which was related to the effect of BRD4 and its target genes in AMPK/ACC mediated fatty acid metabolism.

## Discussion

Although the inhibitors of BRD4 inhibited CALU1 and HT1080 cells in a dose-dependent manner (Fig. S11), it was able to rescue the ferroptosis induced by erastin. In this study, we observed that the loss of BRD4 impinges on mitochondrial function, leading to erastin-ferrop insensitivity. Mechanically, BRD4 assembles SEs with HMGB2 to the promoter regions of HADH, ACSL1, and ACAA2 to promote fatty acid metabolism and maintain sensitivity to ferroptosis.

Based on the effect of BRD4 inhibitors on ferroptosis, some scholars have suggested different views. Among these views is that since antioxidant genes such as GPX4 are downstream target genes of BRD4, inhibiting BRD4 makes cells more sensitive to ferroptosis by further curbing the antioxidant system [24]. However, data from this study suggests that BRD4 does not affect ferroptosis by regulating GPX4. A similar result was observed in previous studies where the cysteine deprivation-induced [25] and the energy stress- induced ferroptosis insensitivity [26] were found to be unrelated to GPX4. A plausible explanation is the heterogeneity of BRD4 regulatory targets in different tumour types.

Previous study have reported that BRD4 plays a crucial regulatory role in various mitochondrial DNA diseases and mitochondrial homoeostasis [27, 28]. As ferroptosis requires the participation of mitochondria [1], it is possible to speculate that BRD4 may affect ferroptosis through the mitochondria-depended way.

For one hand, mitochondria are not only energy factories in cells, but also determine the sensitivity of a range of programmed cell death, such as apoptosis [29], ferroptosis [30], and cuprotosis [31]. Besides, tricarboxylic acid cycle (TCA) and ETC in mitochondria are considered to be essential for ferroptosis in current studies. Typically, cells that rely on mitochondria for energy generation have considered to be more sensitive to ferroptosis due to the generation of mito ROS and the formation of interconnected redox centre for integrating metabolic signals to produce PUFAs which were conducive to ferroptosis [32]. Here, it is worth mentioning that in the initial screening work of anti-ferroptosis targets of this project, some inhibitors that targeting isocitrate dehydrogenase [NADP] (IDH2) and [Pyruvate dehydrogenase (acetyl-transferring)] kinase isozyme 4 (PDK4), which is involved respectively in ketoglutarate metabolism (in TCA cycle) and coenzyme metabolism (in mitochondria) also showed some anti effect on erastin (Data not shown). This is further evidence that any form of metabolic blockade in mitochondria may decreased sensitivity of erastin-ferrop.

For another hand, as target genes of BRD4 identified in this project, HADH, ACSL1, and ACAA2 are all localized in mitochondria and involved in mitochondrial metabolic process. Specifically, HADH was mitochondrial fatty acid β-oxidation enzyme that catalyzes the third step of the β-oxidation cycle [33, 34], and ACAA2 catalyzes the fourth one (the last step) [35]. Meanwhile, ACAA2, which has substrate preference for PUFAs (palmitoleate, oleate, linoleate, and arachidonate), catalyzes the conversion of long-chain fatty acids to their active form acyl-CoAs for both synthesis of cellular lipids, and degradation via beta-oxidation [36–38]. In this study, we found that the oxidative decomposition and the synthesis of PUFAs were significantly decreased in BRD4 inhibition cells. In addition, the AMPK/ACC signalling pathway, a major cellular regulator modulating lipid metabolism and ferroptosis [26], was responded to the inhibition of BRD4 and its target genes. Therefore, BRD4 seems like to control the fatty acid metabolic system in mitochondria, and by blocking which would be a feasible strategy to aviod erastin-ferrop.

Although BRD4 is required to maintain mitochondrial function and aerobic metabolism, tumour cells gradually evolve to survive independent of mitochondria due to chronic lack of nutrient and oxygen supply. As a result, they may be less sensitive to ferroptosis. In a more profound sense, how to precisely target tumour cells to stimulate ferroptosis while minimizing damage to normal cells may be a future challenge. Fortunately, an increasing number of new technologies are being developed to deal with this problem. For instance, ultrasmall silica columns [39] and nano-enabled photosynthesis [40] have been reported to induce ferroptosis by forming iron incorporation based on the physical structure of silica or constructing an oxygen-rich environment in tumour cells to increase lipid peroxidation, which has better destruction ability for cancer cells that are suffering from starvation or resistant to immunotherapy therapy. Based on their mechanism of action, these techniques to stimulate ferroptosis may be more effective for BRD4-deficient tumour cells because they can partly compensate for the insensitivity of ferroptosis caused by metabolism disorders due to the lack of BRD4 expression. Alternatively, BRD4 inhibitors could be considered in combination with these novel methods mentioned above to achieve more effective killing of tumour cells in which BRD4 is relatively abundant.

Some lipid metabolism enzymes like liver X receptor (LXR), stearoyl-CoA desaturase 1 (SCD1), and long-chain-fatty-acid-CoA ligase 4 (ACSL4) have been reported to affect ferroptosis sensitivity [15, 16]. Meanwhile, BRD4 has been found to play roles in lipid accumulation related diseases like the obesity due to high-fat diet [41] and fatty liver [42]. However, it is not clear how BRD4 regulates lipid metabolism and affects ferroptosis. In this study, we demonstrate that BRD4 controls fatty acid metabolism in an classical epigenetic regulation manner by regulating SEs of its target genes.

SE-lncRNAs significantly contribute to gene expression [23]. However, the systematic identification of SE-lncRNAs and their regulated genes still lacks comprehensive recognition [43, 44]. So we used existing databases to search the SEs with BRD4 binding identified by the ChIP assay in various samples, and then delineated the SEs regions that may be involved in regulating HADH, ACSL1, and ACAA2. Transcription abundances of SE-lncRNAs between genes in the overlap coverage regions were tested and found to be significantly down-regulated after BRD4 inhibition, suggesting that BRD4 may regulate its target mRNA genes by affecting their SEs activities. But then again, only these results are still inadequate, more precise technology and more in-depth research will have to be conducted to assess the specific active SEs affected by BRD4 for genes associated with lipid metabolism and ferroptosis.

The enhancer model, in which DNA folds into loops and bends towards promoter regions, is widely accepted [45]. HMGB2 belongs to HMG (high-mobility group) family [28, 46], a “builder” protein family that binds to DNA minor groove and bends DNA to a degree to avoid energy loss so as to facilitates chromatin transcription [47]. In previous study, HMGB2 was reported to bend DNA and form DNA circles to participate in a variety of biological activities [48]. However, the role of HMGB2 in ferroptosis is still unclear. In this study, we reported the binding relationship between BRD4 and HMGB2 and the transcriptional enhancement of lipid metabolism genes depended on their combination. Perhaps, HMGB2 is needed when BRD4 assembles SEs, which ensures much more higher transcription efficiency of their downstream lipid metabolism-related genes and therefore determines the cell’s sensitivity to ferroptosis.

## Conclusion

BRD4 is an essential epigenetic regulator of fatty acid metabolism-related genes that affect ferroptosis. Inhibiting BRD4 paralysed mitochondrial function and reduced lipid ROS formation, making the cells insensitive to erastin-induced ferroptosis.

## Outlook

Since BRD4 is the utmost potential anti-tumour target, more than ten drugs targeting BRD4 have recently entered clinical trials. Therefore, early attention to the relationship between BRD4 and ferroptosis will help clinics design meaningful ferroptosis interventions in advanced cancers. Alternatively, while metabolism is a complete and unified network with complex regulatory mechanisms, this is simply a probable fatty acid metabolic mechanism that BRD4 controls. Therefore, future studies could focus on the metabolism in ferroptosis studies and explore more possible mechanisms that will help the deep understanding of both processes.

## Supplementary information

Fig.S1

Fig.S2

Fig.S3

Fig.S4

Fig.S5

Fig.S6

Fig.S7

Fig.S8

Fig.S9

Fig.S10

Fig.S11

Additional Supplementary Material File

aj-checklist

## Data Availability

All data generated or analyzed during this study are included in this article. The datasets used and/or analyzed during the current study are available from the corresponding author on reasonable request.
