# Supplementary figures and images for: Bromodomain-containing protein 4 (BRD4) as an epigenetic regulator of fatty acid metabolism genes and ferroptosis
Source: Cell Death Dis. 2022 Oct 29;13(10):912. doi: 10.1038/s41419-022-05344-0 (PMC9617950; doi:10.1038/s41419-022-05344-0)

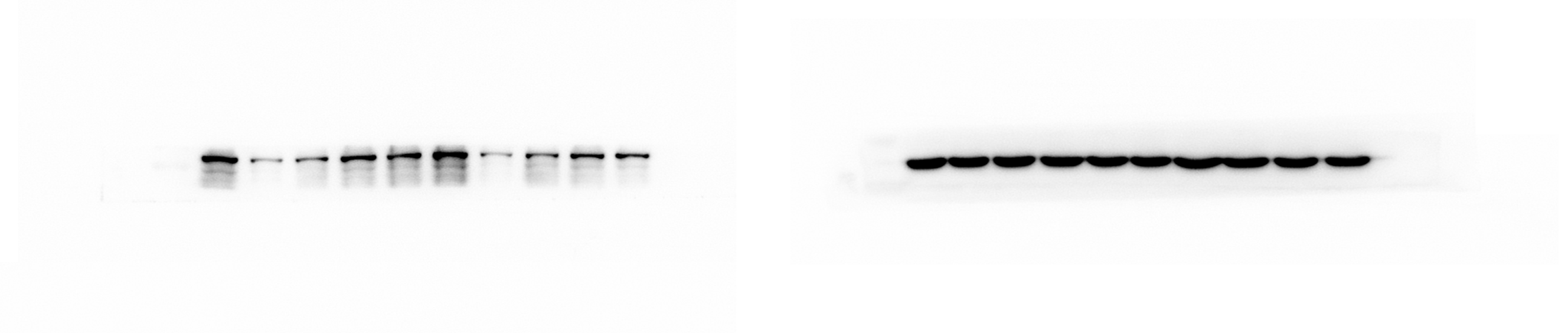

Supplement: Supplementary file 1 — Fig.S1 [file 41419_2022_5344_MOESM1_ESM.tif]

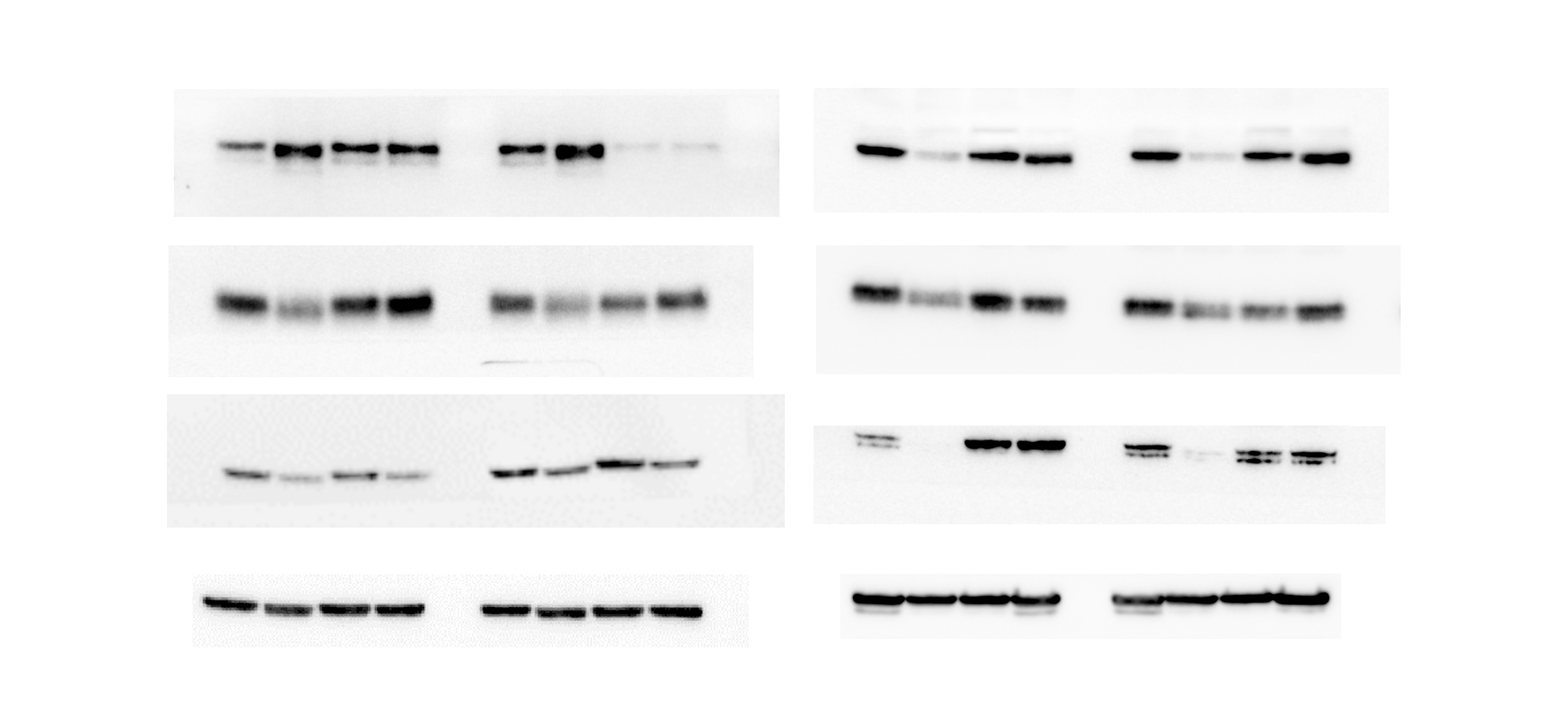

Supplement: Supplementary file 2 — Fig.S2 [file 41419_2022_5344_MOESM2_ESM.tif]

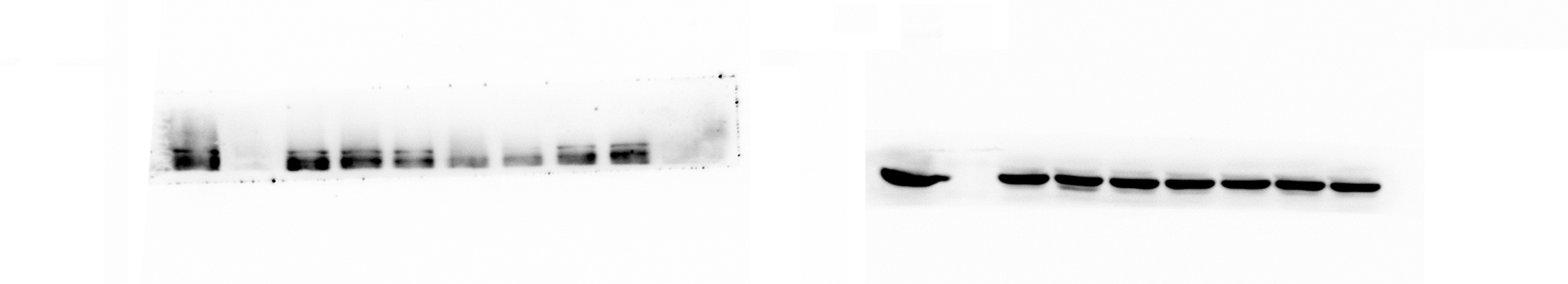

Supplement: Supplementary file 3 — Fig.S3 [file 41419_2022_5344_MOESM3_ESM.tif]

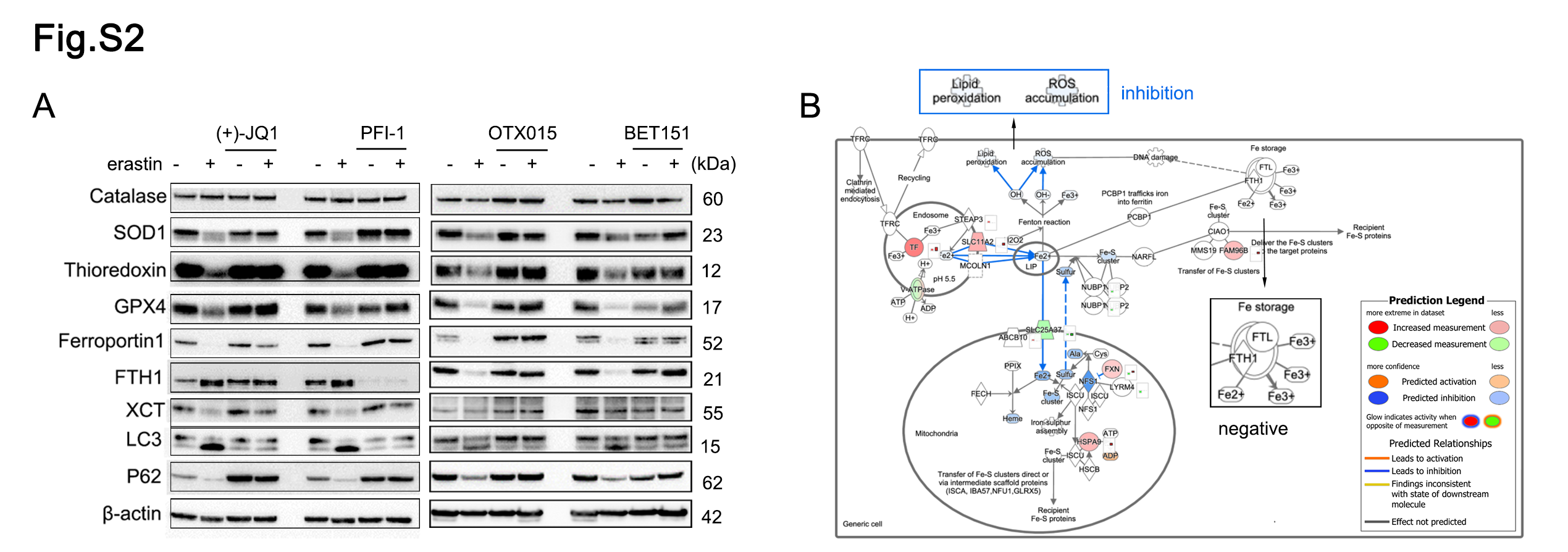

Supplement: Supplementary file 4 — Fig.S4 [file 41419_2022_5344_MOESM4_ESM.tif]

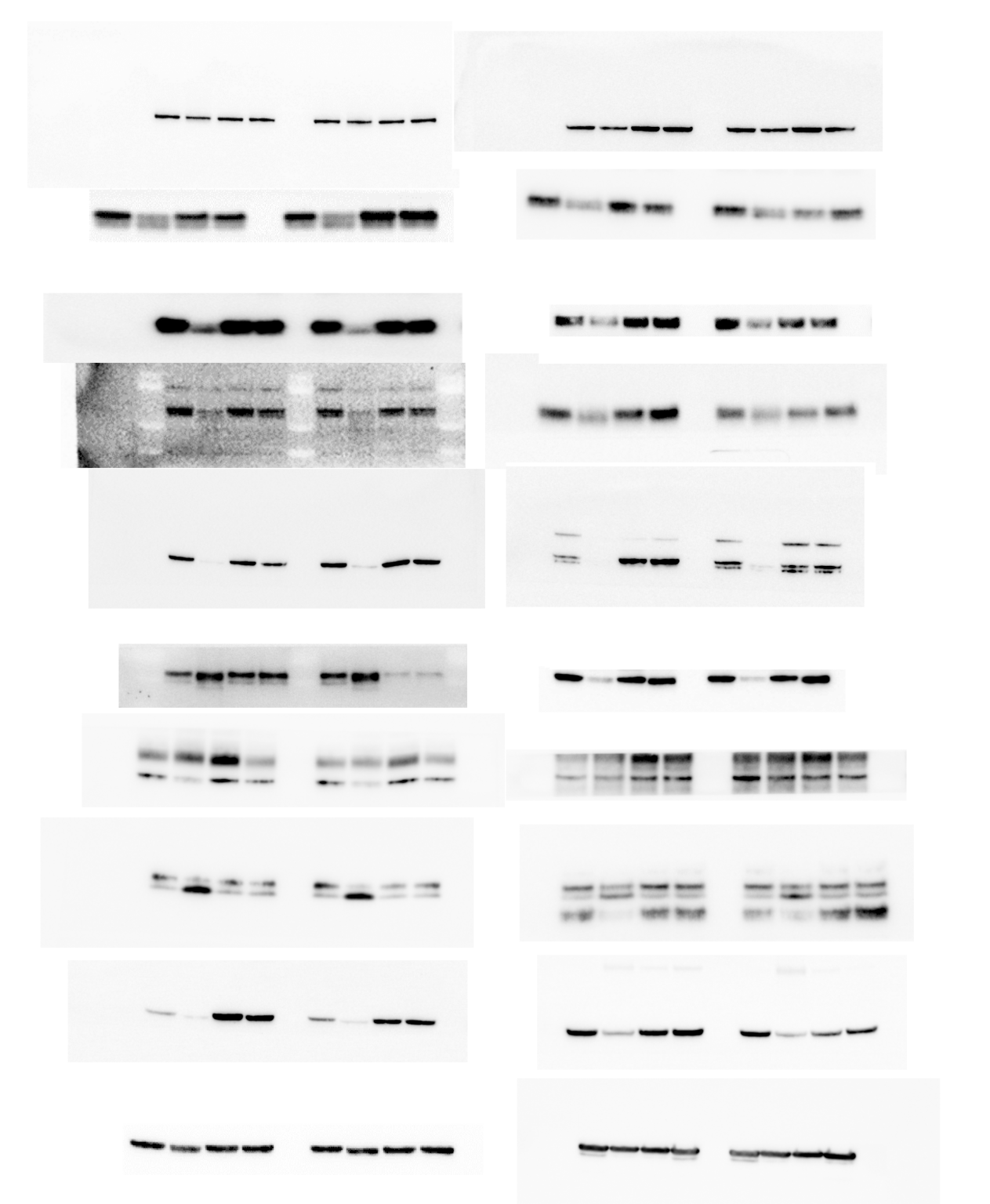

Supplement: Supplementary file 5 — Fig.S5 [file 41419_2022_5344_MOESM5_ESM.tif]

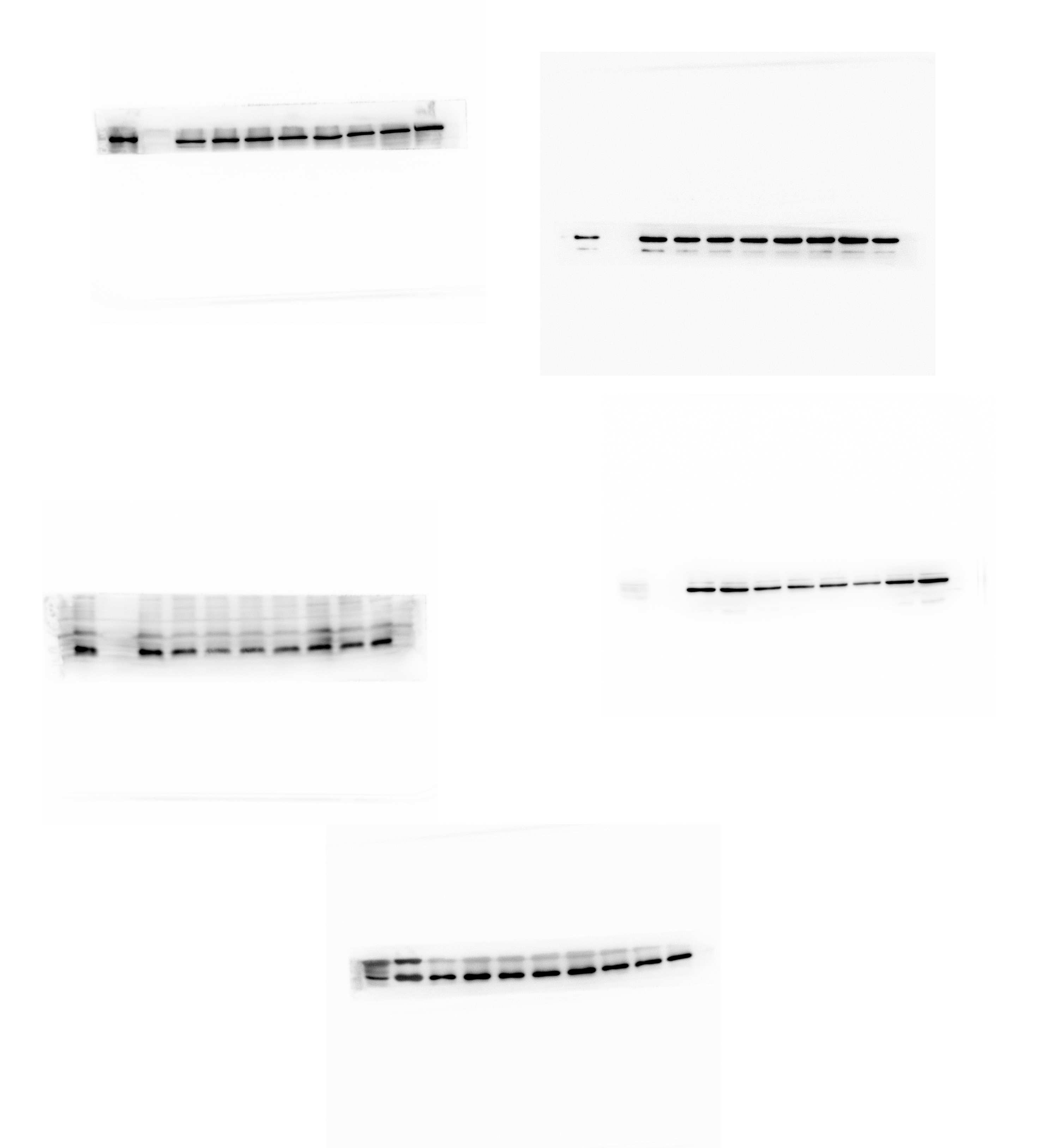

Supplement: Supplementary file 6 — Fig.S6 [file 41419_2022_5344_MOESM6_ESM.tif]

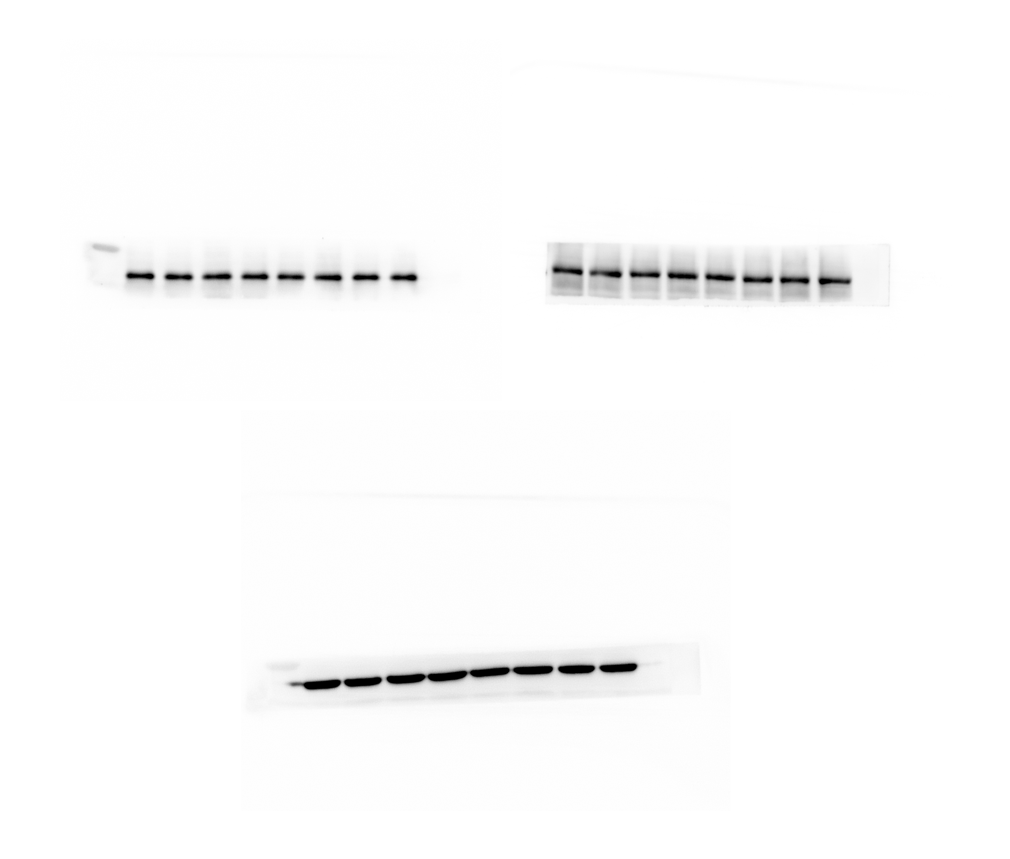

Supplement: Supplementary file 7 — Fig.S7 [file 41419_2022_5344_MOESM7_ESM.tif]

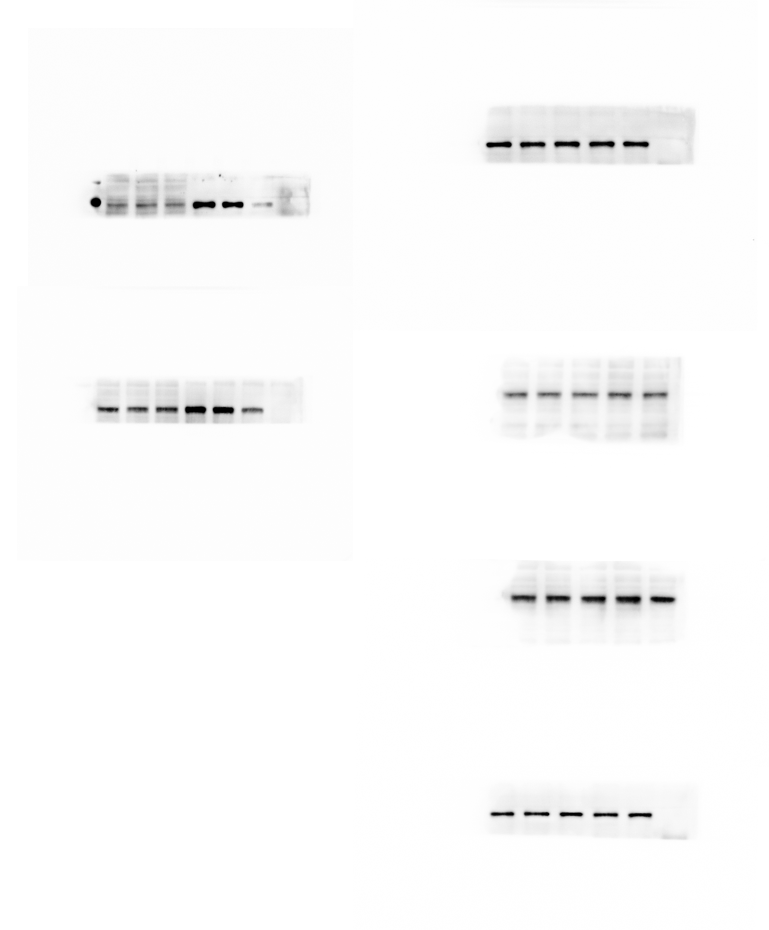

Supplement: Supplementary file 8 — Fig.S8 [file 41419_2022_5344_MOESM8_ESM.tif]

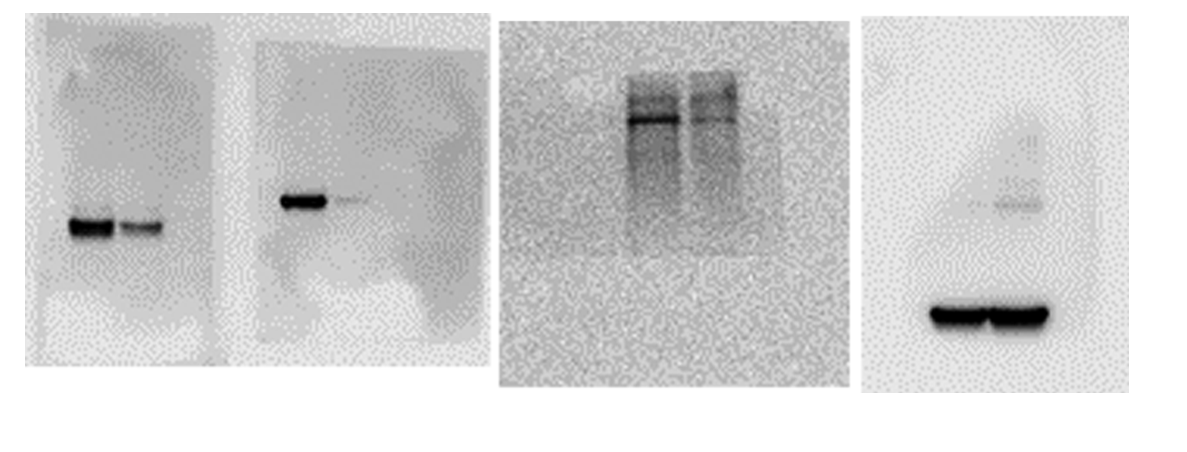

Supplement: Supplementary file 9 — Fig.S9 [file 41419_2022_5344_MOESM9_ESM.tif]

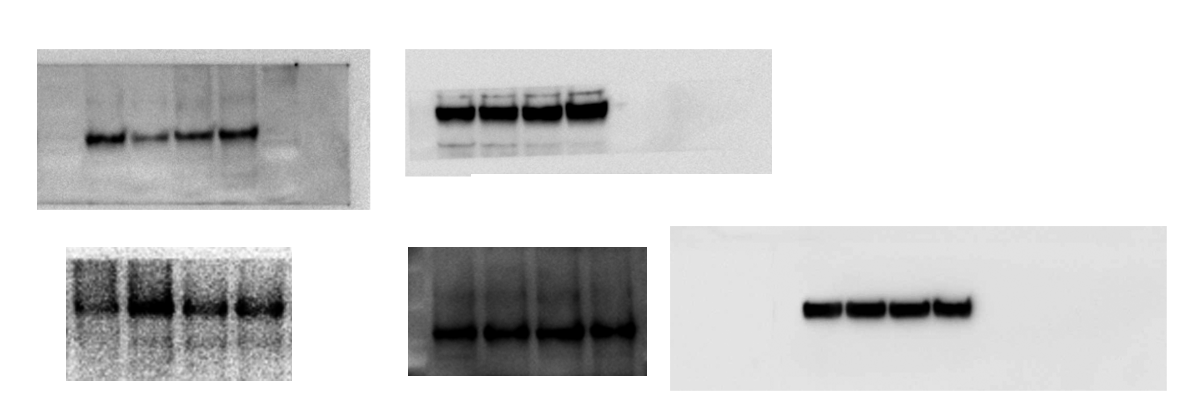

Supplement: Supplementary file 10 — Fig.S10 [file 41419_2022_5344_MOESM10_ESM.tif]

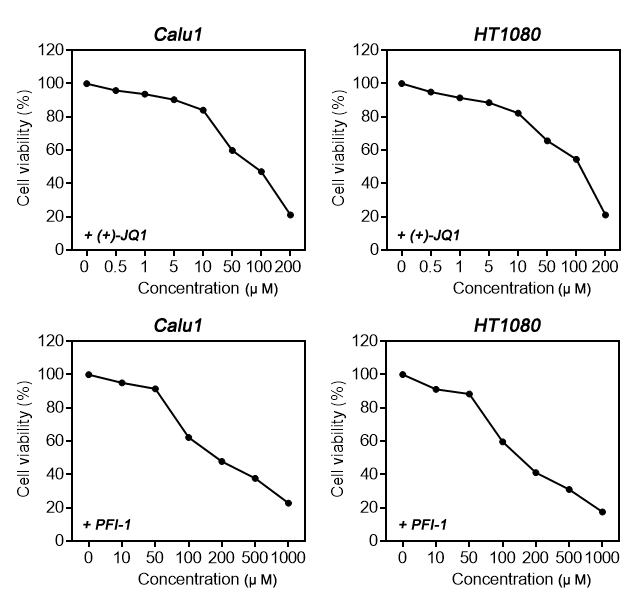

Supplement: Supplementary file 11 — Fig.S11 [file 41419_2022_5344_MOESM11_ESM.tif]
