# Supplementary material for: Bromodomain-containing protein 4 (BRD4) as an epigenetic regulator of fatty acid metabolism genes and ferroptosis
Source: Cell Death Dis. 2022 Oct 29;13(10):912. doi: 10.1038/s41419-022-05344-0 (PMC9617950; doi:10.1038/s41419-022-05344-0)
Supplement: Supplementary file 12 — Additional Supplementary Material File [file 41419_2022_5344_MOESM12_ESM.docx]

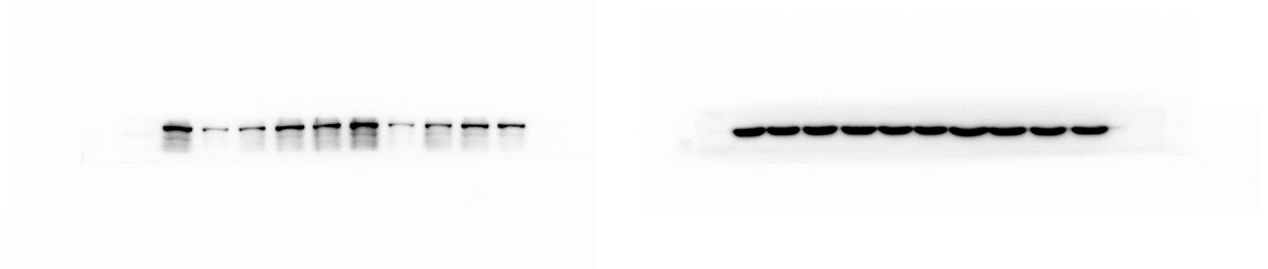


**Fig.S1 Supplementary information of western blots in Fig.2A.**


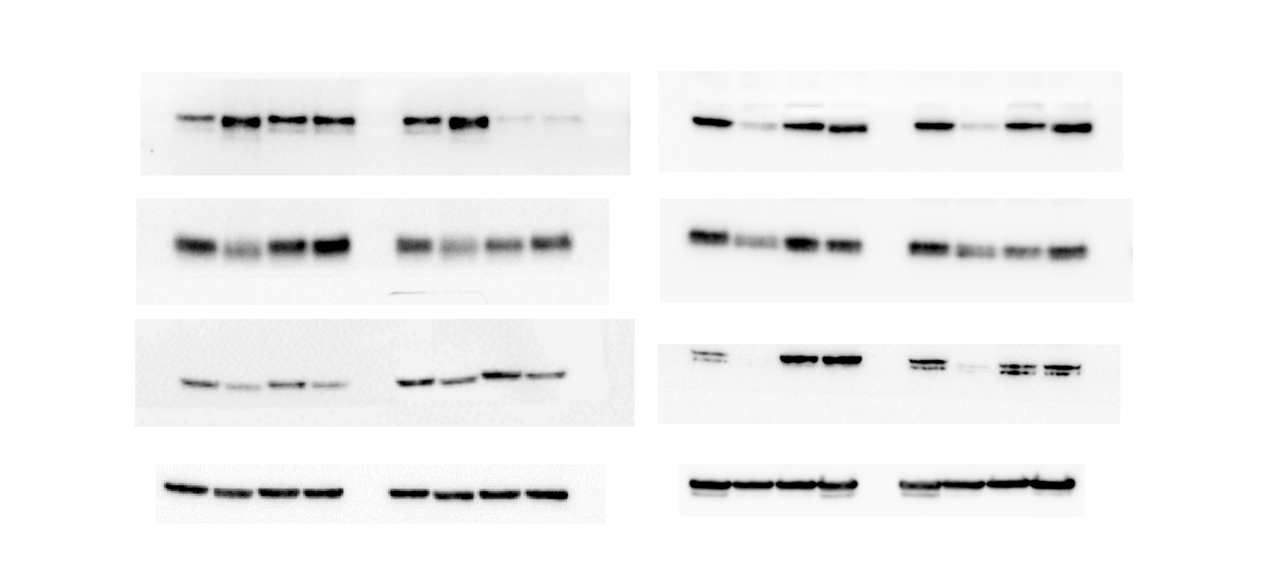


**Fig.S2 Supplementary information of western blots in Fig.2C.**


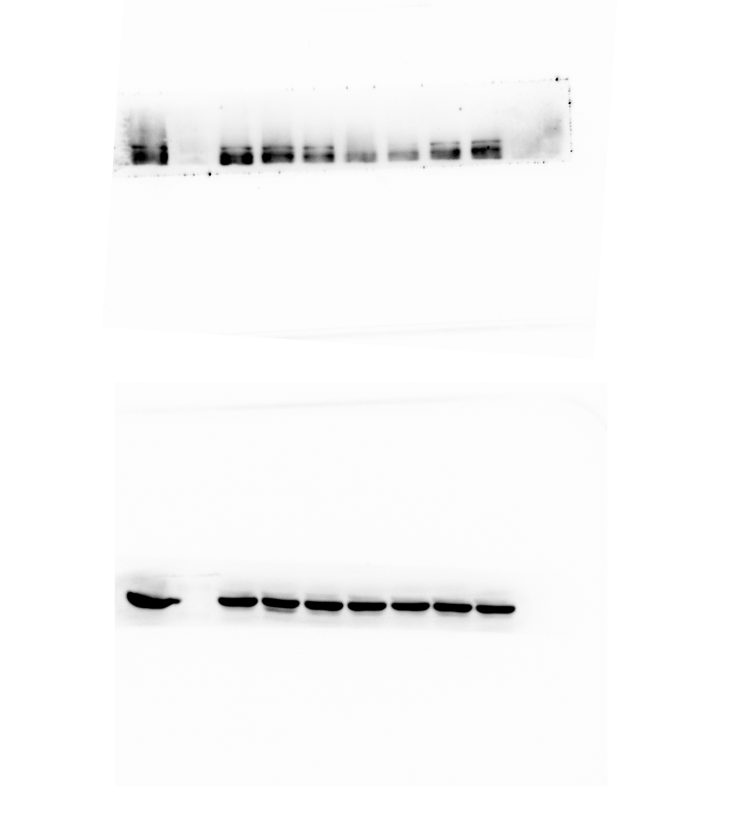


**Fig.S3 Supplementary information of western blots in Fig.2H**


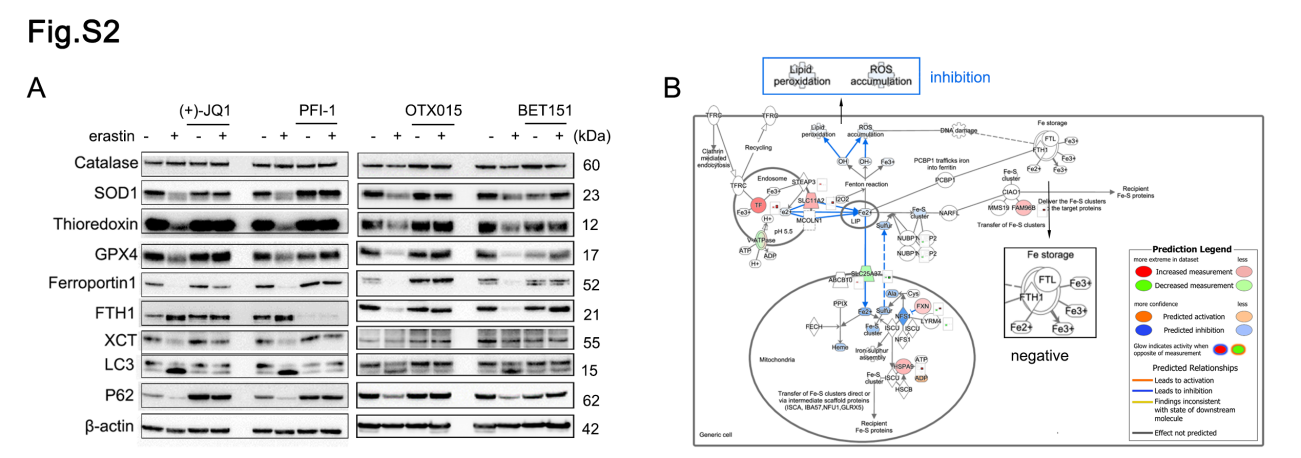


**Fig.S4 The effects of BRD4 inhibitors and erastin on the expression of important molecular proteins in anti-oxidation, autophagy, iron transport, and glutathione synthesis pathways.** (A) Protein expression levels tested by western blotting (n=3, Mean±SD, Tukey-Kramer test of one-way ANOVA, *: P < 0.05; **: P < 0.01; ***: P < 0.005). (B) *Ingenuity Pathway Analysis* (IPA) showing significantly down regulated genes involved in iron metabolism pathways when BRD4 was inhibited with (+)-JQ1. Although the inhibition of BRD4 did not cause significant changes in iron storage-related pathways (black box), it inhibited lipid peroxidation and ROS accumulation pathways (blue box). The IPA signalling pathways were derived from the *Qiagen* IPA software.


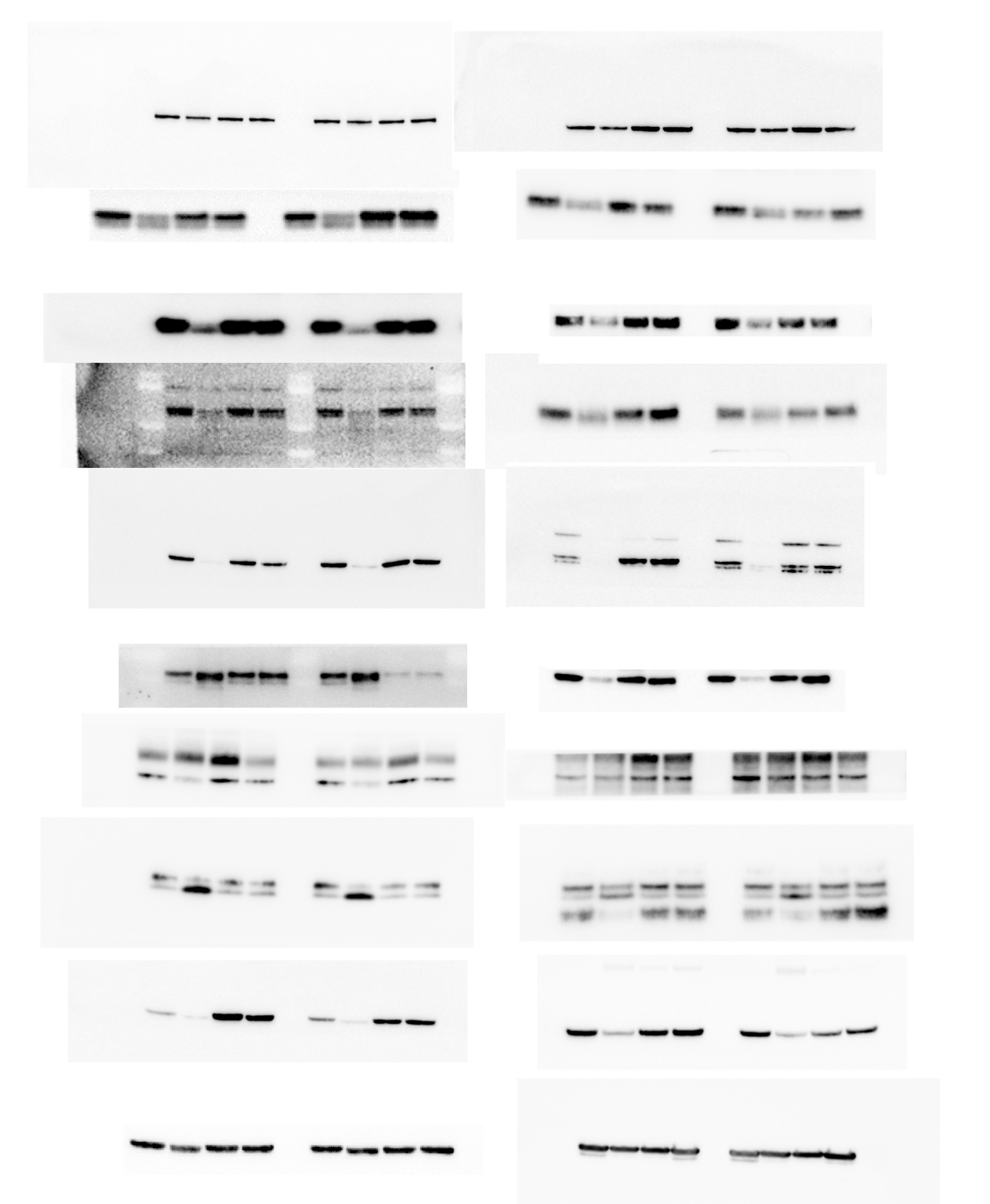


**Fig.S5 Supplementary information of western blots in Fig.S4A**


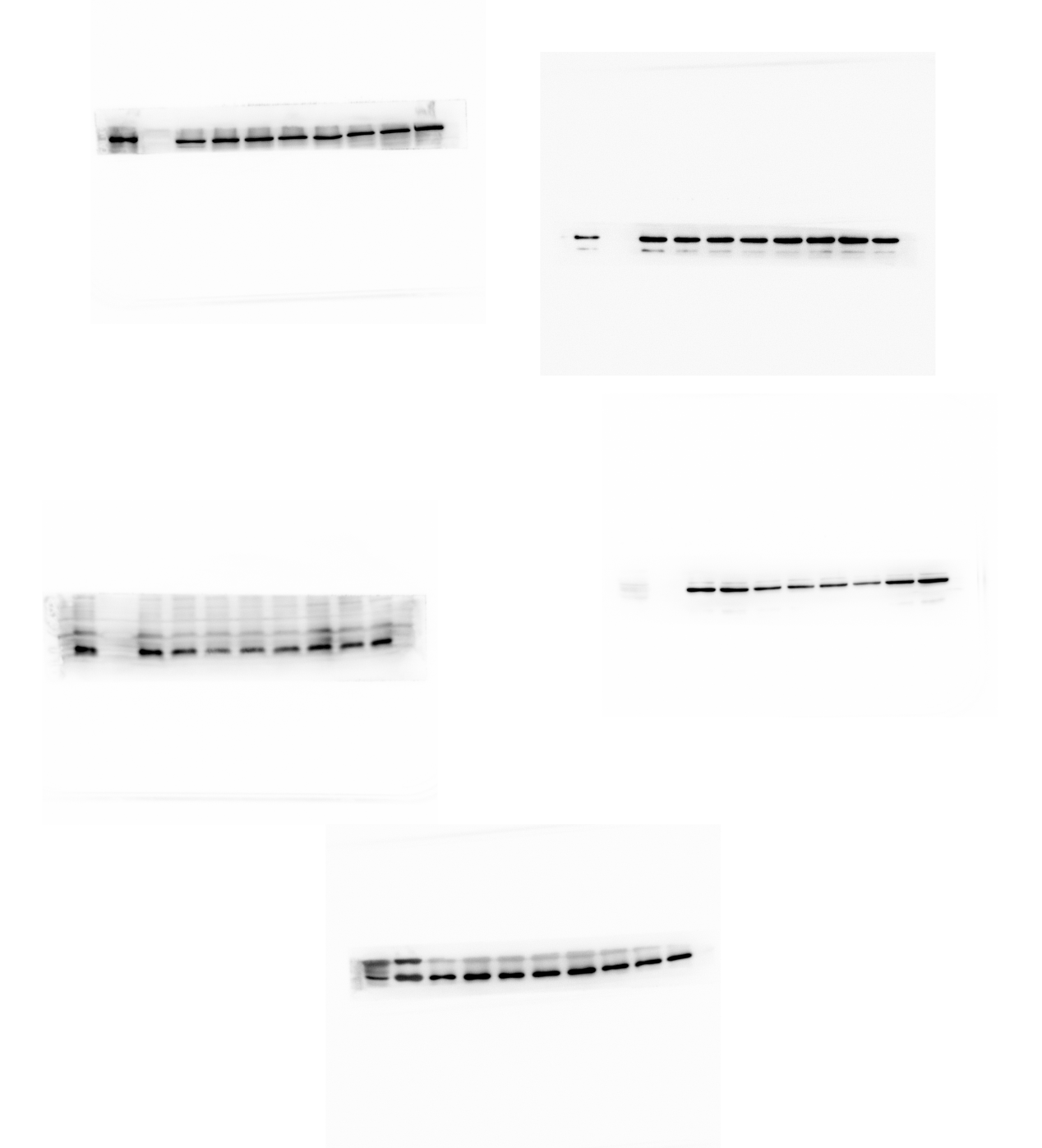


**Fig.S6 Supplementary information of western blots in Fig.4C**

**
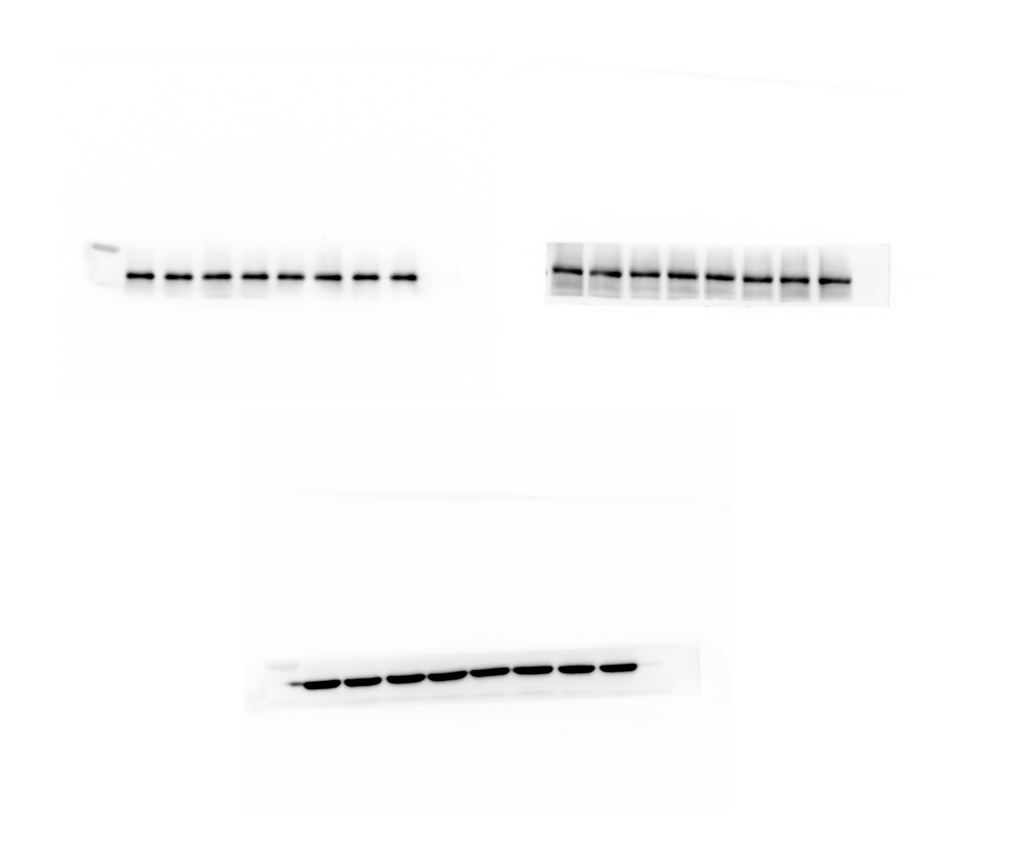
**

**Fig.S7** **Supplementary information of western blots in Fig.9E**


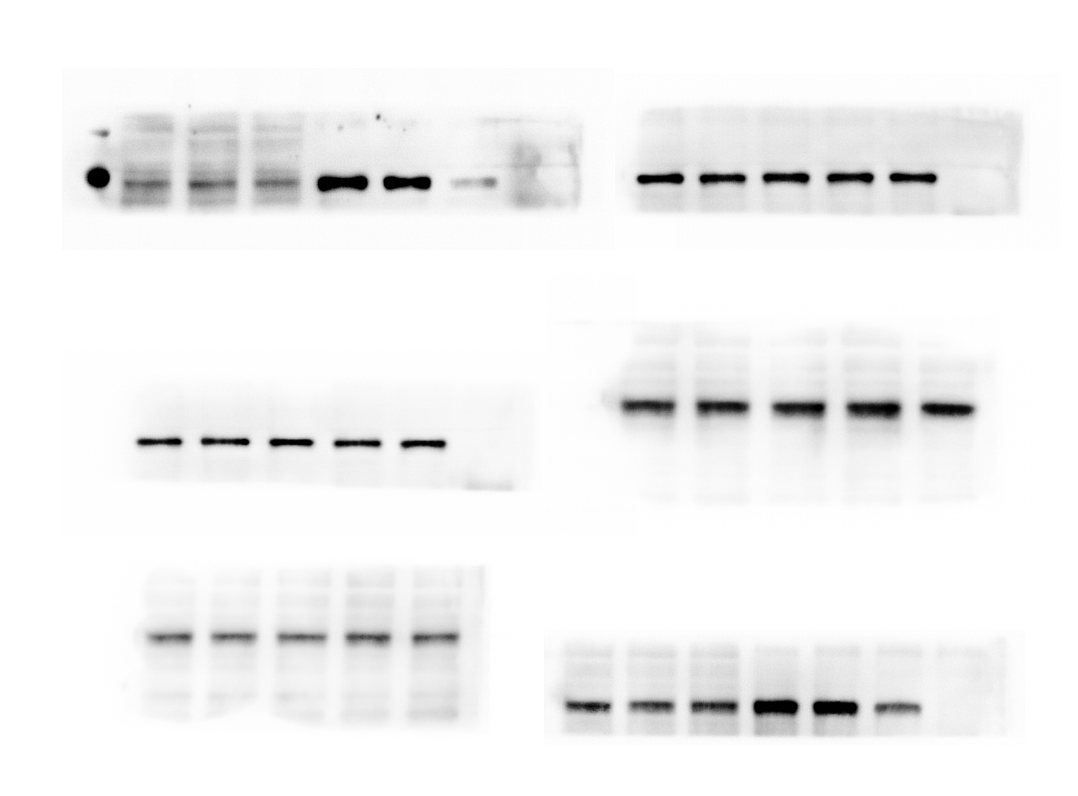


**Fig.S8 Supplementary information of western blots in Fig.9F**


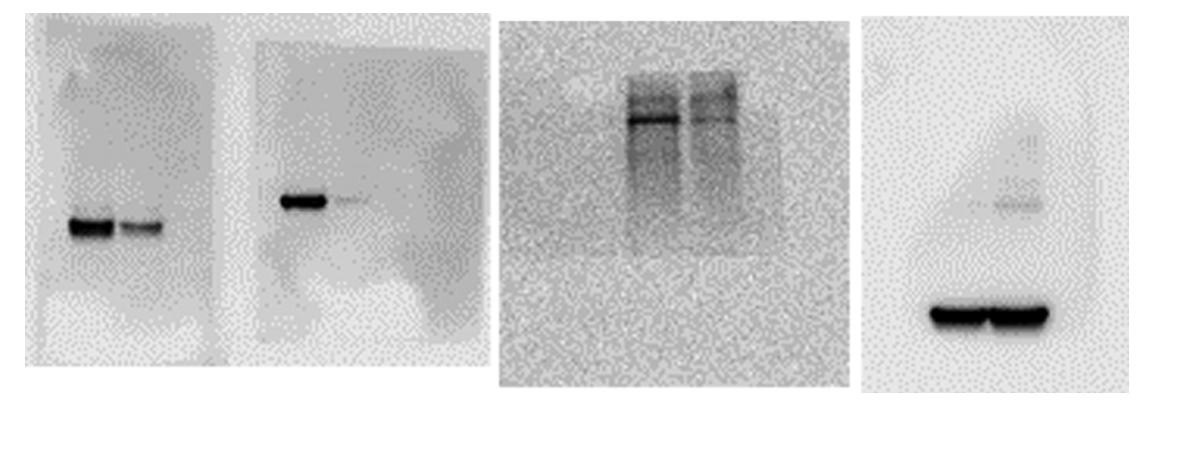


**Fig.S9** **Supplementary information of western blots in Fig.10B.**

**
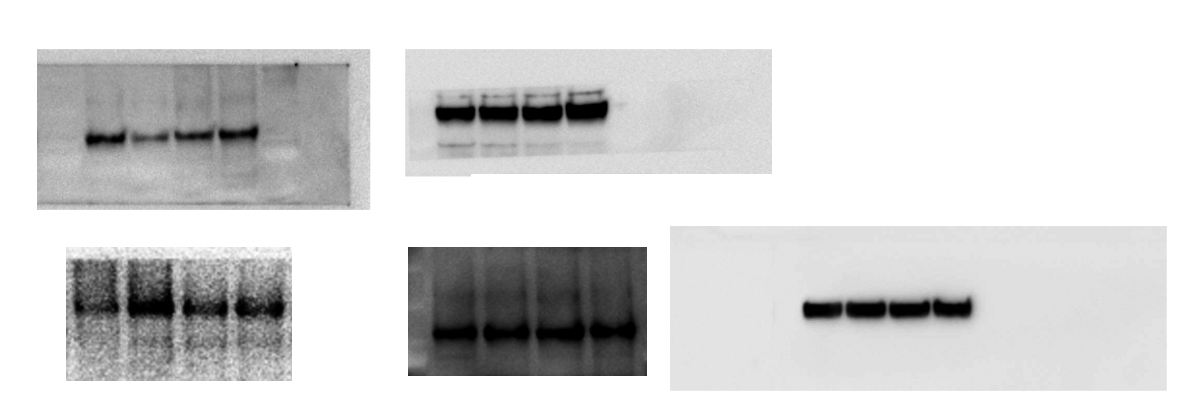
**

**Fig.S10 Supplementary information of western blots in Fig.10F.**


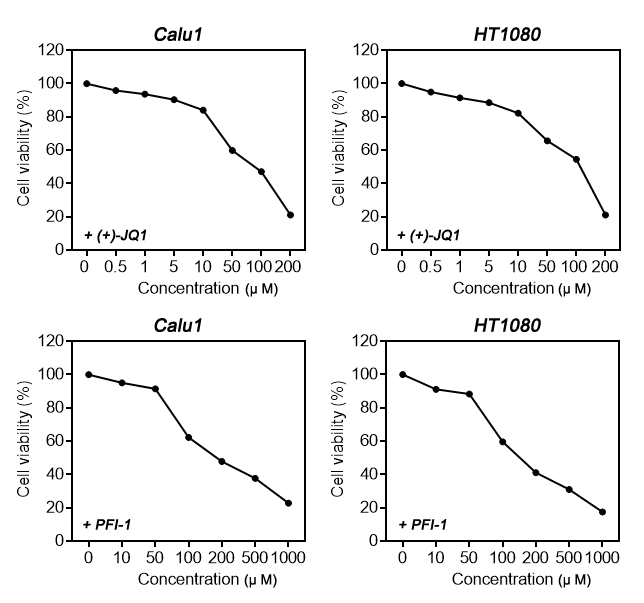


**Fig.S11 Effect of BRD4 inhibitors on cell viability tested by MTT**
